# Gut-microbiome composition, single nucleotide polymorphisms, and differentially expressed genes correlate with aggression in an ant

**DOI:** 10.1016/j.isci.2026.117164

**Published:** 2026-08-24

**Authors:** Patrick Krapf, Francesco Cicconardi, Martin Schilling, Gerhard P. Aigner, Thomas Klammsteiner, Manfred Ayasse, Wolfgang Arthofer, Alexander S. Mikheyev, Florian M. Steiner, Birgit C. Schlick-Steiner

**Affiliations:** 1 Molecular Ecology Group, Department of Ecology, Universität Innsbruck, 6020 Innsbruck, Austria; 2 Institute for Biodiversity and Ecosystem Dynamics, University of Amsterdam, 1090 GE Amsterdam, the netherlands; 3 Organismal and Evolutionary Biology Research Programme, University of Helsinki, 00560 Helsinki, Finland; 4 School of Biological Sciences, University of Bristol, Bristol BS8 1TQ, UK; 5 Department of Genetics and Evolutionary Biology, Institute of Biosciences, University of São Paulo, São Paulo, Brazil; 6 Institute of Evolutionary Ecology and Conservation Genomics, Ulm University, 89081 Ulm, Germany; 7 Ecology and Evolution Unit, Okinawa Institute of Science and Technology Graduate University, Onna, Okinawa, Japan; 8 Research School of Biology, Australian National University, Canberra, ACT, Australia

**Keywords:** whole-genome sequencing, transcriptomics, gut microbiome, cuticular hydrocarbons, behavior, start of aggression, tetramorium alpestre

## Abstract

Animals frequently display aggressive behavior, for example, when competing for food. Aggression is influenced by various extrinsic and intrinsic factors such as the microbiome and genetics. However, we currently lack understanding what factors cause animals to start aggression. Here, we use an ant species to test if chemical, microbiome, genomic, and/or transcriptomic traits correlate with the start of aggression and the reactions to it, namely reacting aggressively or peacefully. We found nine bacterial operational taxonomic units, mutations in two genes, and eight differentially expressed genes, which were positively or negatively associated with the start of aggression or reactions to it. The microbiome and genetic factors are mainly linked to hormone signaling and neurological and synaptic functions, respectively. The results indicate that multiple traits, possibly acting in concert, may affect the start of aggression and reactions to it. We speculate that such traits could promote aggression and play important evolutionary roles.

## Highlights

• We tested if traits correlate with the start of aggression and reactions to it
• Nine bacterial operational taxonomic units were linked to hormone signaling
• Mutations in two genes and eight differentially expressed genes were detected
• Both were linked to synaptic functions—suggesting they could promote aggression

## Graphical abstract

**Figure:**
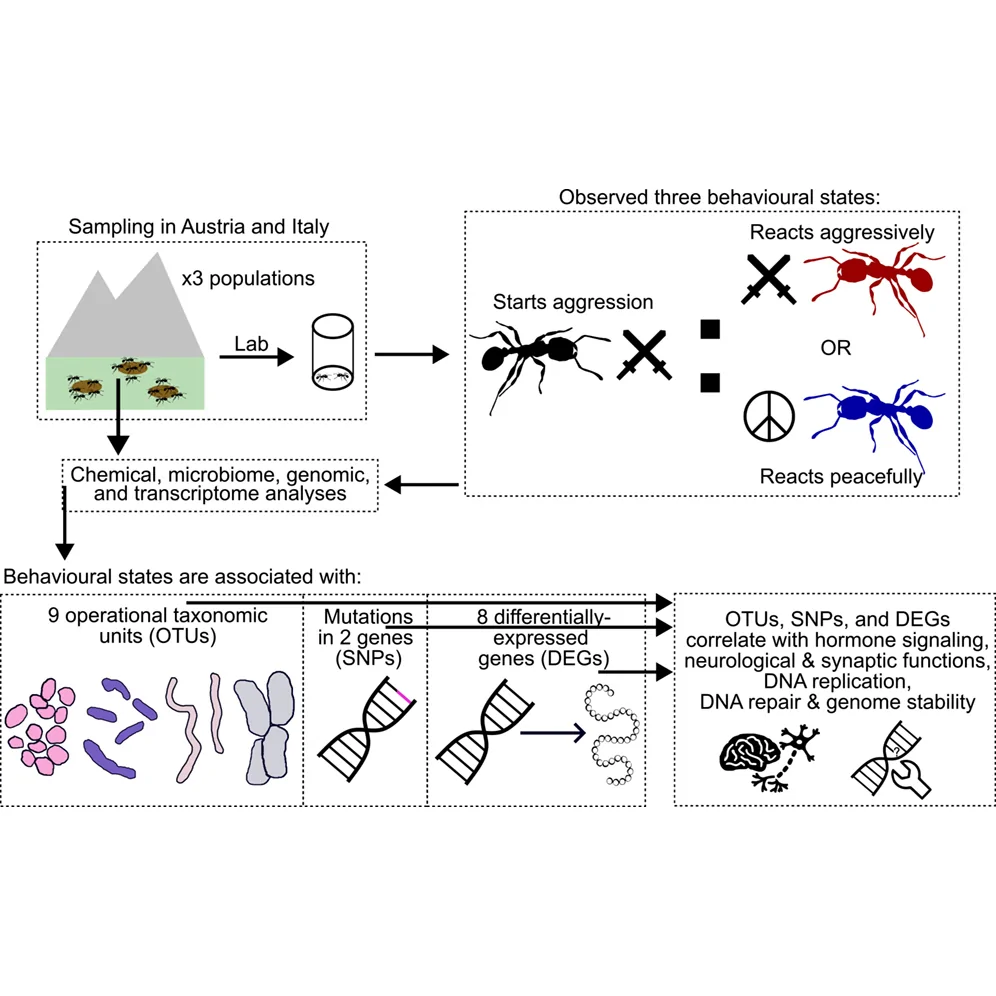

## Teaser

Genomics; Microbiome; Zoology

## Introduction

Aggressive behavior among individuals of the same species is a frequently observed behavior in animals.^1^ It is a vital aspect of animals’ fitness and survival and often context-dependent.^2^^,^^3^ For example, it can occur during food or mate competition, territory defense, and offspring protection against predators.^4^ Such adaptive aggression^3^^,^^5^ can lead to increased fitness. For instance, winners of fights can consume more or higher-quality food or obtain mates for reproduction.^1^ However, aggression can incur harms such as stress and energy or time costs. At its worst, it can also be deadly^6^ by increasing the risk of injuries and/or exposure to predators.^7^

Various extrinsic and intrinsic factors can lead to aggression. Extrinsic factors are, among others, higher ambient temperature and can lead to increased aggression in humans and animals.^8^^,^^9^ Intrinsic factors such as experience (i.e., repeated stimuli such as winning aggressive encounters),^10^ neurochemical factors (i.e., changes in serotonin, dopamine, or octopamine),^3^ or differentially expressed genes (DEGs)^3^ influenced by the gut microbiome can also promote aggression.^11^^,^^12^^,^^13^

Despite these promising insights, our understanding of the underlying mechanisms that lead to the start of aggression (i.e., when two individuals meet and one starts aggressive behaviors such as fighting) is limited. Nevertheless, some drivers are known: for example, individual experience,^14^^,^^15^ previous experience in winning a fight,^16^ or recognizing another individual^17^ can affect whether an individual starts aggression. In particular, animals such as insects use chemical cues^18^ (cuticular hydrocarbons; CHCs) to recognize and attack enemies.^19^ Besides experience and recognition, the microbiome,^11^^,^^12^^,^^13^^,^^20^^,^^21^^,^^22^ genetic changes (e.g., mutations in genes^23^^,^^24^), and/or DEGs (e.g., in neuronal or synaptic functions^17^) may also affect whether individuals start aggression.

Ants are known for their aggressive behavior. For example, California harvester ants (*Pogonomyrmex californicus*) often fight for over 30 min, and such fights often result in fatal outcomes with one or both workers dying.^25^ On the other end of this spectrum are “peaceful” ants, which frequently refrain from fighting individuals from different colonies of the same species. Peaceful behavior is less frequently observed, but is known from several species such as *Lasius austriacus*,^26^ *Lasius flavus*,^27^ and *Tetramorium alpestre.*^9^ However, even in such predominantly peaceful species, aggression can be observed, leading to the unresolved question of what factors lead to the start of aggression.^28^^,^^29^

**Figure 1:**
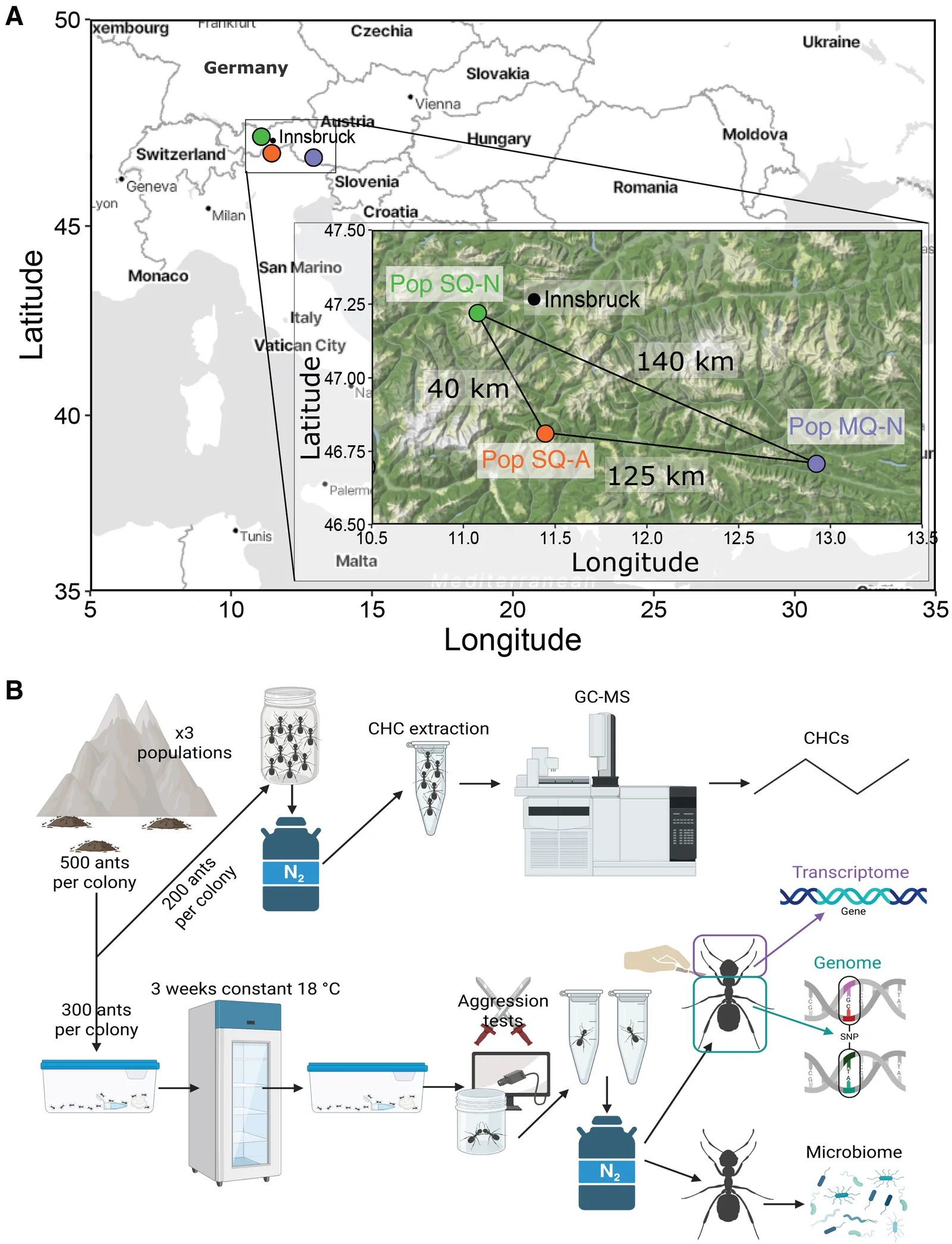
Sampling map and schematic overview of the assays (A) Sampling area in Central Europe. The populations were defined based on preliminary data and aggression assays conducted in this study: population SQ-N in green colors represents single-queened, non-aggressive colonies. Population SQ-A in orange represents single-queened, aggressive colonies. Population MQ-N in blue represents multiple-queened, non-aggressive, potentially supercolonial colonies. The inset in (A) shows the three populations in closer detail and the linear distances between all three populations. (B) Schematic overview of the assays, created in BioRender by Krapf, P. (2025), https://BioRender.com/ecq4a2x. Note: N_2_ = nitrogen; CHC = cuticular hydrocarbons; GC-MS = gas chromatography-mass spectrometry.

Here, we used the high-elevation ant species *T. alpestre* to test whether chemical, microbiome, genomic, and/or transcriptomic traits correlate with the start of aggression in ants, specifically workers. This species displays a behavioral continuum ranging from aggression to peacefulness.^9^^,^^30^^,^^31^ We collected workers from three colonies each from three previously described populations.^9^^,^^30^^,^^31^ They either comprise single-queened and aggressive colonies “SQ-A”, single-queened and non-aggressive colonies “SQ-N”, or multiple-queened and non-aggressive colonies “MQ-N” (i.e., supercolonies consisting of multiple colonies connected over a large area,^32^ N_col_ = 9; Figures 1A and 1B; Table S1; note, no multiple-queened and aggressive colonies are known within this species). We conducted recognition (own colony against alien colony) and aggression assays and selected individual worker ants that displayed either of the following behavioral states, *started aggression*, *reacted aggressively*, or *reacted peacefully* for chemical, microbiome, genomic, and transcriptomic analyses (Figure 1C). We then integrated results from these analyses in a final multinomial logistic regression to assess their joint impact on the behavioral states.

## Results

### Aggression tests and selection of workers for whole-genome and -transcriptome sequencing

**Figure 2:**
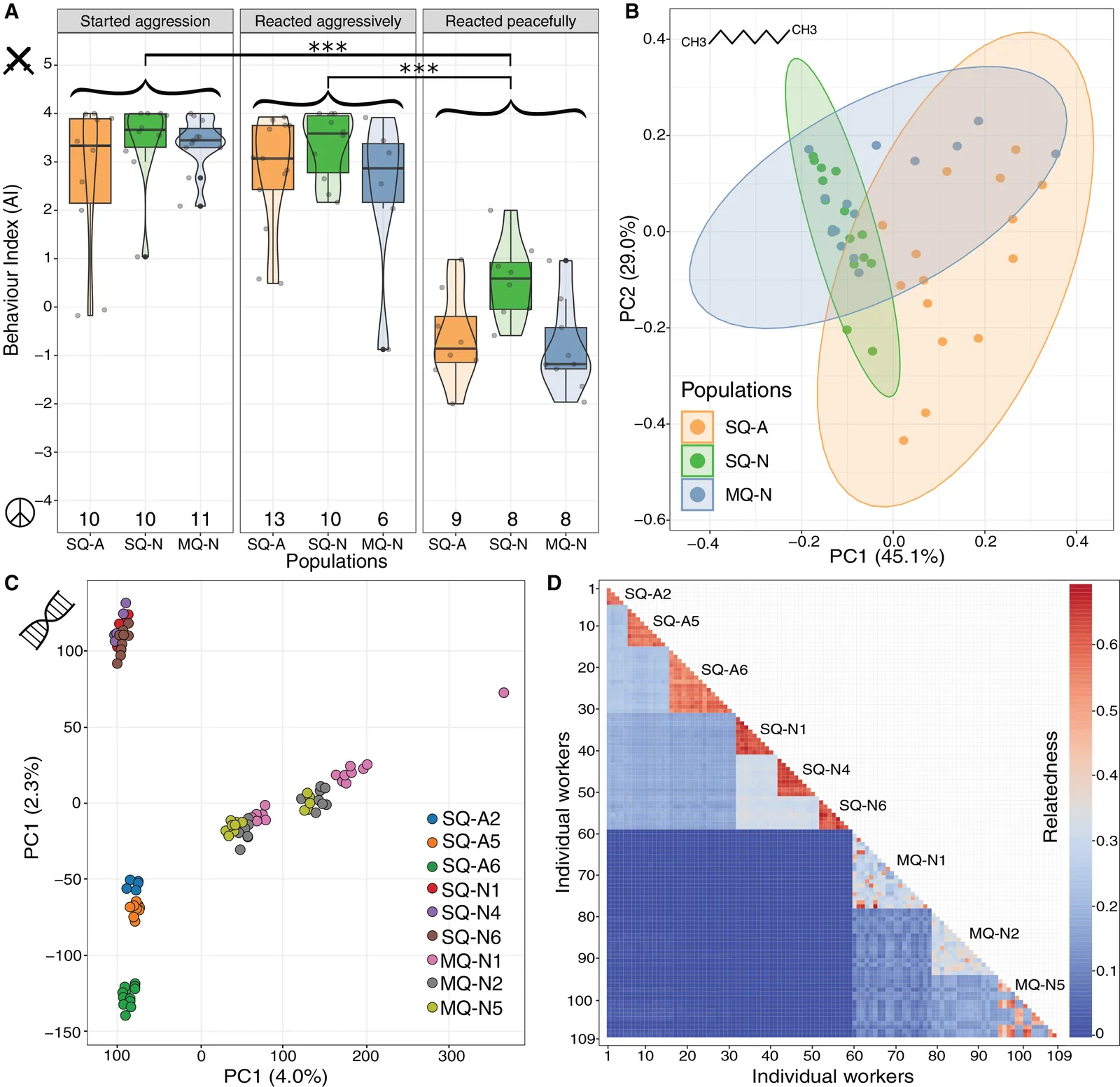
Results of the aggression tests, PCA of the CHCs, PCA of the LD-pruned SNPs, and within- and between colony relatedness (A) Combined boxplots, violin-, and scatterplots displaying the three behavioral states for the three populations along the behavior index *AI*, which denotes aggressive (5–1), neutral (0), and peaceful (−4 to −1) behavior. Whiskers in boxplots represent upper and lower 25% of the data, the box represents 50% of data and the line the median. The width in the violin plots represents the density of the data at specific values, while the light gray points represent the observed values. Behavioral data from workers from of each of the three behavioral states *started aggression*, *reacted aggressively*, and *reacted peacefully* were first combined, and then compared against each other in a pairwise test as indicated by the parentheses. Based on the respective behaviors, we selected workers for whole-genome and whole-transcriptome sequencing. Numbers above colony names represent the sample size for each group. Only workers used in the transcriptomic data are displayed. Note: SQ-A = single-queened and aggressive colonies, SQ-N = single-queened and non-aggressive colonies, MQ-N = multiple-queened and non-aggressive colonies. ∗∗∗ = *p* < 0.001 by ANOVA and Tukey honest significant post-hoc test. (B) PCA using 63 cuticular hydrocarbon (CHC) compounds that were found in all analyzed workers (*N*_workers_=44). The three populations SQ-A, SQ-N, and MQ-N differ significantly in their CHC bouquet on the first axis (PC1: 45.1%) by pairwise.adonis test. However, no cluster or complete separation is apparent. (C) PCA of linkage-disequilibrium (LD)-pruned SNPs from whole-genome sequence data (*N*_workers_=109). The first axis (4.0%) separates the supercolonial population MQ-N from the aggressive and non-aggressive populations SQ-A and SQ-N. The second axis (2.3%) separates the aggressive population SQ-A (bottom-left image of the PCA) from the non-aggressive population SQ-N (upper-left image of the PCA). (D) The within- and between-rest relatedness was calculated following the Manichaikul et al.^33^ relatedness using genomic SNP data (*N*_workers_=109). Each square represents a pairwise comparison between all samples. A dark red color of a square indicates a close relatedness between two samples, while a dark blue color indicates a distant/loose relatedness.

To select workers for whole-genome and transcriptome sequencing that displayed either of the three behaviors, *started aggression*, *reacted aggressively*, and *reacted peacefully*, we conducted standardized one-on-one worker aggression tests^9^ among all nine colonies. We analyzed the behavior of each individual worker and calculated a behavior index. By conducting an analysis of variance (ANOVA), we found that the behavior differed among the behavioral states (Figure 2A; ANOVA: df = 2, F-value=78.02, *p* value < 0.001). To confirm that peaceful behavior has lower aggression values, we pairwise compared the behavioral states of all workers from each behavioral type using a Tukey honest significant test: workers that *started aggression* and ones that *reacted aggressively* had significantly higher aggression values throughout the confrontations than workers that *reacted peacefully* (*started aggression* vs. *reacted peacefully*, *p* value < 0.001; *reacted aggressively* vs. *reacted peacefully*, *p* value < 0.001). However, workers that *started aggression* and ones that *reacted aggressively* had similar aggression values (*started aggression* vs. *reacted aggressively*, *p* value = 0.597). The within-colony behavior (control; not shown) did not reveal any aggression. Additionally, workers preferred own odors over alien odors or a control (for details, see the section “recognition assays” in the supplemental results). Based on the aggression tests and ANOVA, we selected 85 and 109 workers for whole-transcriptome and whole-genome sequencing, respectively.

### Cuticular hydrocarbon analysis

The cuticular hydrocarbon (CHC) bouquet differed significantly among populations. We found 78 compounds in the odor bouquets (hydrocarbon chain length C12 to C35; GC-MS analyses of CHC-extracts of five workers pooled per colony, except for one colony with four workers; N_workers_=44). From these 78 CHC compounds, 63 were found in all workers tested (Table S2). We visualized the CHC compounds multidimensionally (PCA, Figure 2B) and conducted a permutational analysis of variance and pairwise tests (PERMANOVA, vegan package^34^; pairwise.adonis package^35^). We found that CHC compounds differed significantly between the three populations (overall analysis: R^2^ = 0.32, df = 2, pseudo-F statistic = 9.62, *p* value < 0.001; SQ-A vs. SQ-N: R^2^ = 0.37, df = 1, pseudo-F statistic = 15.93, *p* value = 0.001; SQ-A vs. MQ-N: R^2^ = 0.26, df = 1, pseudo-F statistic = 9.72, *p* value = 0.001; SQ-N vs. MQ-N: R^2^ = 0.11, df = 1, pseudo-F statistic = 3.30, *p* value = 0.011). In more detail, colonies from the single-queened and aggressive population SQ-A (colonies SQ-A2, SQ-A5, SQ-A6) overlapped with CHC compounds of colonies from the single-queened and non-aggressive population SQ-N (SQ-N1, SQ-N4, SQ-N6) and from the multi-queened and non-aggressive population MQ-N (MQ-N1, MQ-N2, MQ-N5), but CHC compounds of population MQ-N overlapped the most. Using the CHC compound data, we conducted a hierarchical cluster analysis and found that CHC extracts from SQ-N and MQ-N were more similar to each other and partially clustered together (Figure S1), which was also observed by pairwise comparison. In contrast, samples from SQ-A5 were more similar to colonies from populations SQ-N and MQ-N than to SQ-A2 and SQ-A6 colonies.

### Whole-genome and whole-transcriptome analyses

Observed heterozygosity and pairwise genomic differentiation were similar among samples, but relatedness was higher in multiple-queened and non-aggressive colonies. After quality checks and filtering, 184,145 and 69,191 single nucleotide polymorphisms (SNPs) were kept in whole-genome and whole-transcriptome VCF files, respectively (109 and 82 samples, respectively). The mean observed heterozygosity for whole-genome samples was 0.30 (min = 0.23, max = 0.48) and for whole-transcriptome samples 0.15 (min = 0.001, max = 0.37). The pairwise genomic differentiation values (Weir-Cockerham F_ST_) were very similar across populations with 0.005 for populations SQ-A:SQ-N, 0.004 for SQ-A:MQ-N, and 0.008 for SQ-N:MQ-N. Visualized multidimensionally (linkage disequilibrium-pruned PCA with DNA samples; Figure 2C), the multiple-queened and non-aggressive population MQ-N separated from the other two single-queened populations SQ-A and SQ-N. Samples from population SQ-N clustered together, while colonies from population SQ-A appeared well separated. Samples from population MQ-N clustered together regardless of colony identity. Mean within-colony relatedness was slightly higher in populations SQ-A and SQ-N than in population MQ-N (SQ-A: 0.57, SQ-N: 0.63; MQ-N: 0.33; Figure 2D; Table S3). The colony queen number (estimated using the relatedness values) was approximately one in all colonies of populations SQ-A and SQ-N and at least two in all colonies of population MQ-N (Table S3).

We identified three SNPs associated with the behavioral states using a genome-wide efficient mixed model association (GEMMA) analysis. We assessed associations between SNPs as well as insertions/deletions (“InDels”) and three SNPs (henceforth SNP1-3; Figure S2). SNP1 is in the sequence of the gene called “mediator of RNA polymerase II transcription subunit 26” (located at scaffold 11, site 2457277). SNP2 is in the sequence of an unknown gene (located at scaffold 165; site 28,302). SNP3 is in the sequence of the gene “*gastrulation-defective*” (*gd*; located at scaffold 185, site 110,741).

**Figure 3:**
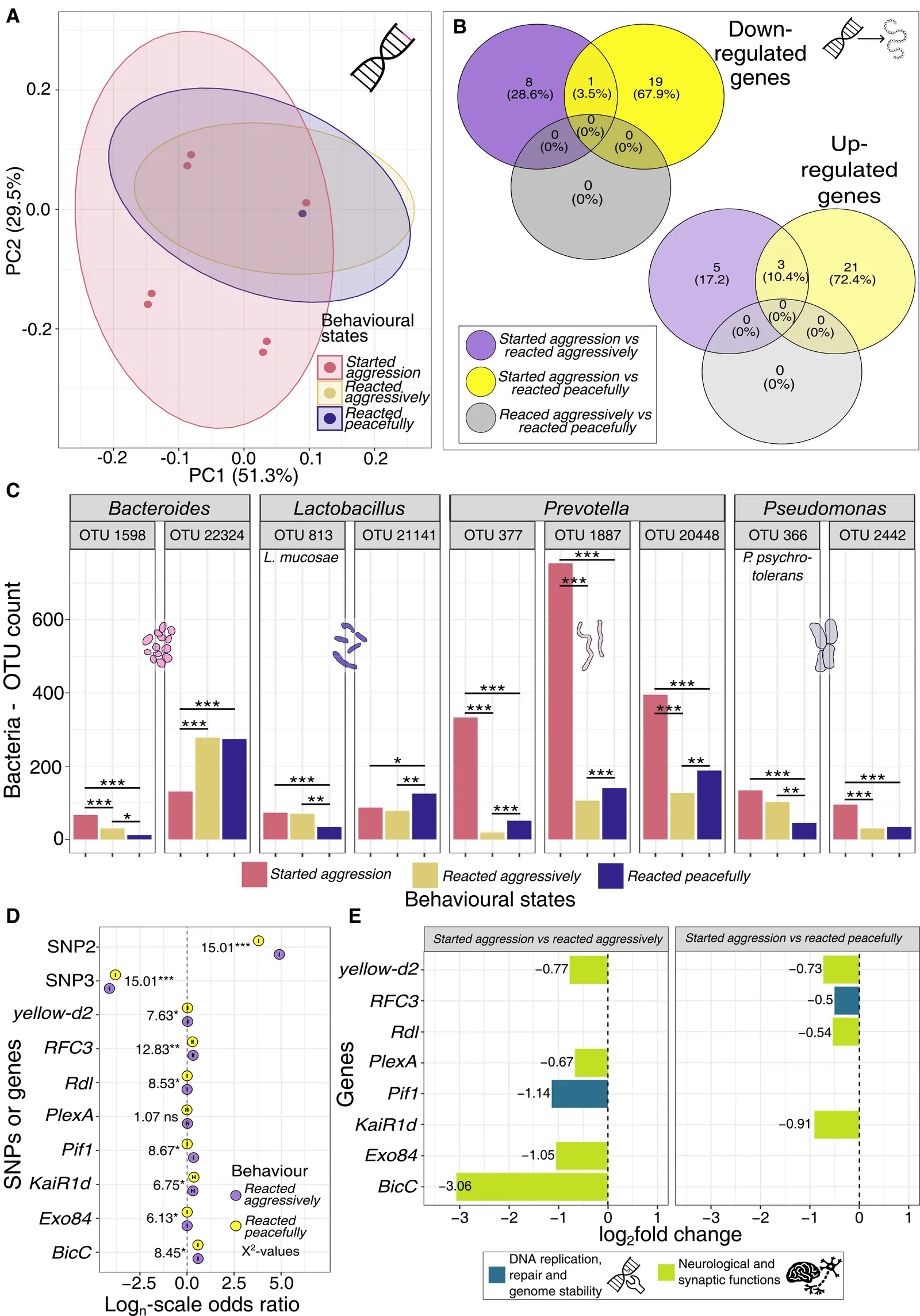
PCA of the allelic states of the SNPs, Venn diagrams of the DEGs, bacterial OTU counts, results of the multinomial logistic regression, and log_2_fold change of the DEGs (A) Principal component analysis (PCA) of the genomic single nucleotide polymorphisms (SNP) states from the three genes that were above the suggestive line in the genome-wide efficient mixed model association (GEMMA) analysis (see also Manhattan Plot, Figure S4, *N*_workers_=109). In the PCA, individuals that are homozygous for the reference allele of the respective genes are represented as 0/0 and individuals that are heterozygous for the reference alleles are 0/1. The three behavioral states *started aggression*, *reacted aggressively*, and *reacted peacefully* are colored in red, yellow, and purple, respectively. In the PCA, the behavioral state *started aggression* displays allelic combinations that are not observed in the other two behavioral states. (B) Venn diagrams of, in total, 57 differentially expressed genes (DEGs) which were significantly down and upregulated (FDR-corrected for multiple testing with < 0.05; *N*_workers_=109) with known gene names based on Flybase (https://flybase.org/, retrieved 10.04.2025). Dark colors represent downregulated genes and light colors upregulated genes. (C) Counts of nine operational taxonomic units (OTU) that were associated with the behavioral states in the multinomial logistic regression (*N*_workers_= 49). Significant differences in the counts are represented with asterisks, ∗∗∗ = < 0.001; ∗∗ = < 0.01; ∗ = < 0.05 by generalized linear model and pairwise comparisons (“emmeans”). (D) Results of the multinomial logistic regression displaying the logistic odds ratio values for each SNPs and DEGs separately including the Chi-squared and *p* values from the likelihood ratio test (LRT) to assess the global significance of the SNPs or DEGs on the behavior (*N*_workers_=82). Significant differences in the counts are represented with asterisks, ∗∗∗ = < 0.001; ∗∗ = < 0.01; ∗ = < 0.05 by a two-tailed Wald *t* test with *started aggression* as baseline. (E) Log_2_fold change for the DEGs that we identified as significant in the multinomial logistic regression shown for the comparison of *started aggression* vs. *reacted aggressively* and *started aggression* vs. *reacted peacefully* (*N*_workers_=82). The comparison *reacted aggressively* vs. *reacted peacefully* did not yield any significant up or downregulated genes and no data are now shown.

The allelic states of the genomic SNPs differed between the behavioral states. We visualized the homozygous and heterozygous allelic states for the three behavioral states multidimensionally (PCA; Figure 3A). The behavioral state *started aggression* revealed a larger variation and had slightly different allelic states in the SNPs compared with the other two behavioral states. We assessed if the count of the reference or alternative alleles for each specific SNP was different across rows (Pearson’s Chi-squared test for count data with simulated *p* value and 2000 Monte Carlo replicates and subsequent post-hoc test). For SNP1, 36 out of 41 (88%) workers that *started aggression*, 35 out of 39 workers (90%) that *reacted aggressively*, and 26 out of 29 (90%) workers that *reacted peacefully* were homozygous for the reference allele (Figure S3; Table S4). For SNP1, the allele counts were not different across behavioral states (Figure S3, one-SNP1; χ^2^ = 0.09, *p* value = 1.000). For SNP2, 18 out of 41 (43%) workers that *started aggression* were homozygous in the SNP state, while 2 out of 39 (5%) workers that *reacted aggressively* and 2 out of 29 (7%) workers that *reacted peacefully* were homozygous in the SNP state. More individuals that *started aggression* were homozygous for the reference allele (SNP2: χ^2^ = 22.98, *p* value < 0.001; see Table S4 for pairwise comparisons). For SNP3, 30 out of 41 (73%) workers that *started aggression* were heterozygous for the SNP state, 10 out of 39 (26%) workers that *reacted aggressively*, and 11 out of 29 (38%) workers that *reacted peacefully* were heterozygous for the behavioral state. More individuals that *started aggression* were heterozygous for the reference allele (SNP3: χ^2^ = 19.381, *p* value < 0.001).

### Differential gene-expression analyses

We identified several differentially expressed genes (DEGs) associated with the behavioral states (N_workers_=82). We pairwise compared all behavioral states (i.e., *started aggression* (N_AntsSeq_ = 31), *reacted aggressively* (N_AntsSeq_ = 28), and *reacted peacefully* (N_AntsSeq_ = 23). In each comparison, we found ∼17,000 DEGs or isoforms, of which roughly 100 were significant (for details on the comparisons, see the section “DEGs” in the supplemental results). In the comparison between ants that *started aggression* and *reacted aggressively*, we found 13 significantly upregulated genes and 36 downregulated genes in workers that *started aggression* (false-discovery rate (FDR)-corrected for multiple testing; Figure S4A, volcano plot—red dots). When comparing ants that *started aggression* with workers that *reacted peacefully*, we found 28 and 61 genes that were significantly up- and downregulated, respectively. In the comparison between ants that *reacted aggressively* and *reacted peacefully,* no gene was significantly up or downregulated in workers that *started aggression*.

When comparing the behavioral states *started aggression* vs. *reacted aggressively* with *started aggression* vs. *reacted peacefully*, we found 30 upregulated and 28 downregulated genes that were found in both (< 0.05-corrected genes with known functions were used; Figure 3B). Specifically, the genes were upregulated or downregulated in the behavioral state *started aggression*. For the 30 upregulated genes, five genes were exclusively found in the comparison *started aggression* vs. *reacted aggressively* (*CG3800*, *CG3902*, *CDase*, *Rhp*, and *Moe*; Table S5), 22 genes exclusively in the comparison *started aggression* vs. *reacted peacefully* (*CG34367*, *CG13625*, *CG3655*, *apolpp*, *CG14687*, *CG3655*, *Gat*, *Sur-8*, *mRpL9*, *CG9175*, *CG6656*, *Phm*, *Socs16D*, *Vav*, *CG3860*, *CG32225*, *CG9426*, *alph*, *CG16974*, *CG10483*, *AP-2alpha*, and *bchs*; Table S5), and three in both comparisons *started aggression* vs. *reacted aggressively* and *started aggression* vs. *reacted peacefully* (*CG3061*, *svr*, and *Syt4*; Table S5). The log_2_fold changes of these genes ranged between 0.12 and 3.19 for *started aggression* vs. *reacted aggressively* and between 0.11 and 0.50 for *started aggression* vs. *reacted peacefully* (Table S5). No gene was found to be differentially expressed in the comparison *reacted aggressively* vs. *reacted peacefully*.

For the 28 downregulated genes, eight genes (*BicC*, *Pif1* (*CG3238)*, *Exo84*, *PlexA*, *Tret1-1*, *Taf5*, *Doa*, and *Vps35*; Table S5; Figure 3B) were found in the comparison *started aggression* vs. *reacted aggressively*, 19 in the comparison *started aggression* vs. *reacted peacefully* (*CG3822*, *Rdl*, *RFC3*, *CG10431*, *Cdep*, *Dscam1*, *CG7492*, *CG6910*, *snRNP-U1-70K*, *CG31550*, *l(1)G0196*, *CG9346*, *CG32486*, *agt*, *Gcn5*, *baz*, *CG13366*, *U2af50*, and *CG8108*; Table S5), and one (*yellow-d2*; Table S5) was found in both comparisons *started aggression* vs. *reacted aggressively* and *started aggression* vs. *reacted peacefully*. The log_2_fold changes of these genes ranged between −3.06 and −0.15 for *started aggression* vs. *reacted* *aggressively* and between −1.60 and −0.15 for *started aggression* vs. *reacted peacefully* (Table S5). Of these genes, two were highly expressed: gene *CG3800* was highly upregulated (log_2_fold change = 2.48) and gene *BicC* was highly downregulated (log_2_fold change = −3.06). All the aforementioned genes were used for the multinomial regression analyses (for details, see section below “analyzing multiple data layers jointly”).

### Analysis of high-throughput 16S rRNA gene sequencing data

To assess whether the laboratory maintenance affected the microbiome and whether the microbiome (i.e., bacteria and archaea) is associated with the three behavioral states, 16S rRNA gene sequencing was conducted with 49 workers from the populations SQ-A and SQ-N, and 16 additional “control” samples from SQ-A and SQ-N (i.e., directly frozen in the field and not used in aggression tests; for details, see the STAR Methods section). Subsequently, we conducted a principal coordinate analysis (PCoA) with these samples. Laboratory maintenance did not change the bacterial operational taxonomic units (OTUs) composition in the ants (Figure S5A). Also, the behavioral states were mixed with control samples (Figure S5B). Only samples from one colony (SQ-N6) were separated from the other samples on the first axis.

Four bacterial genera were frequently found across the dataset. From an average of 116,096 ± 20,583 raw reads per sample, 79,844 ± 19,799 quality-filtered reads per sample remained, subsampled to an equal depth of 37,808 reads (N_workers_=49). After rarefaction, 22,215 unique OTUs were identified, including 264 archaea, 19,773 bacteria, and 2178 unknown OTUs. We excluded OTUs not classified at the genus level. Of the remaining OTUs, the genera *Pseudomonas* (8.7% relative abundance), *Bacteroides* (6.1%), *Lactobacillus* (5.2%), and *Prevotella* (4.4%) were found most frequently.

Besides these four bacterial OTUs (*Pseudomonas*, *Bacteroides*, *Lactobacillus*, and *Prevotella*), we selected and further calculated the relative frequency for four additional bacterial genera and one order, namely *Acetobacter*, *Enterococcus*, *Fusobacterium*, *Megamonas*, and the order Rhizobiales. These bacteria are also known to affect behavior in humans,^20^^,^^36^ dogs,^12^ *Drosophila*,^11^ and ants.^13^ Of these, the genus *Pseudomonas* was most frequent (25.4%, Table S6) followed by *Bacteroides* (20.7%), *Lactobacillus* (18.6%), *Prevotella* (17.0%), *Enterococcus* (11.3%), *Megamonas* (5.4%), *Acetobacter* (0.7%), *Fusobacterium* (0.5%), and the order Rhizobiales (0.3%). Using these bacterial OTU genera, we selected OTUs that had a frequency of at least 100 across the behavioral states (*N*=119), thus focusing on the most frequent OTUs.

With these 119 OTUs, we conducted a sliding-window approach in a multinomial logistic regression to count how often they were significantly associated with the behavior states. Across these models, the most frequent OTUs (N_OTUs_=58 with a frequency ≥ 10) included the genera *Bacteroides* (25% relative percentage across 58 models), *Lactobacillus* (9%)*, Prevotella* (43%)*, Pseudomonas* (17%), the order Rhizobiales (4%), and the genus *Fusobacterium* (1%). We further reduced the OTU number for downstream analyses yielding 18 OTUs (e.g., using OTUs with a higher or lower frequency than one across the counts of the behavioral states; for details see “analysis of 16S rRNA gene-sequencing data” in the STAR Methods). With these 18 OTUs, we assessed whether OTU counts differed among behavioral states by conducting a generalized linear model and pairwise comparison (“emmeans” package^37^; Tukey corrected for multiple testing).

Nine OTUs were significantly associated with the behavioral states, namely two *Bacteroides* spp., two *Lactobacillus* spp., three *Prevotella* spp., and two *Pseudomonas* spp. (Table S8; Figure 3C). In the two *Bacteroides* species, significantly more OTU counts occurred in the behavioral state *started aggression* and *reacted aggressively* than in *reacted peacefully* (OTU 1598) as well as fewer counts in *started aggression* than *reacted aggressively* or *reacted peacefully* (OTU 22324). For the genus *Lactobacillus*, significantly fewer and more OTU counts occurred in the behavioral state *reacted peacefully* than in *started aggression* or *reacted aggressively* in *Lactobacillus mucosae* and in *Lactobacillus* sp., respectively. In the three *Prevotella* and two *Pseudomonas* species, significantly more OTU counts occurred in the behavioral state *started aggression* than in the state *reacted aggressively* and *reacted peacefully* (OTUs *Prevotella* 377, 1887, 20448; *Pseudomonas* 366, 2442). For the three *Prevotella* species, also more OTU counts occurred in the behavioral state *reacted peacefully* than in *reacted aggressively*.

### Analyzing multiple data layers jointly

We integrated genomic, transcriptomic, chemical, and environmental data layers in 24 multinomial logistic regression models to assess if they were associated with the behavioral states (N_workers_=82). The site-specific environmental variables were calculated manually or extracted from the WorldClim dataset^38^ (for details, see “environmental variables used in the multinomial regression analyses” in the STAR Methods section”). In more detail, we used SNPs, gene-expression counts, within-colony relatedness, site-specific air temperature, the first PC of the CHC analysis, soil nitrogen values, mean annual precipitation, precipitation of the warmest quarter, mean annual temperature, and maximum temperature of the warmest month as explanatory variables (for details, see STAR Methods section “combining SNPs, DEGs, CHCs, relatedness, and environmental variables counts in a multinomial regression”).

We used allelic states, normalized expression counts, and continuous environmental variables as predictors in a single model. We excluded microbiome data because they were not available for the multiple-queen population MQ-N. In the models, we used the behavioral state *started aggression* as the baseline and run an intercept-only model as reference. To find the best model explaining the data, we selected various combinations of explanatory variables resulting in 24 models: models 1–8 used only the four genes that were found in both behavioral comparisons (genes *CG3061*, *svr*, *Syt4*, *yellow-d2*), and models 9–16 and models 17–24 included either all upregulated or all downregulated genes found in the differential gene expression analysis, respectively. We used log likelihood ratio tests to the robustness of the variables.

In the four best-fitting models, we found two SNPs and eight genes that were associated with the behavioral states. The models were model 2, 4, 12, and 20, which included the following SNPs and DEGs: SNP2 and SNP3 (*gastrulation-defective*) and DEGs *yellow-d2*, *BicC*, *Pif1*, *Exo84*, *PlexA*, *KaiR1d*, *Rdl*, and *RFC3* (for detailed results, see the supplemental results section “logistic multinomial regression analyses” and Tables S9–S17). Although SNP1 (sequence of the gene called “mediator of RNA polymerase II transcription subunit 26”) was significant in model 20, we excluded it because it was not significant in the SNPs-only model. We then combined the aforementioned variables (i.e., SNP2, SNP3, *yellow-d2*, *BicC*, *Pif1*, *Exo84*, *KaiR1d*, *RD*, *RFC3*, and *PlexA*; note SNP2 = unknown gene; SNP3 = gene *gastrulation-defective*) in a final multinomial logistic regression. This final model explained significantly more variance than the intercept-only model (Likelihood ratio test of multinomial models, likelihood ratio: 89.73, *p* value < 0.001). A goodness-of-fit measure was calculated by comparing the fit of observed and expected values, and the model displayed a good fit (χ^2^ = 74.89, df = 4; *p* value < 0.001, residual deviance = 89.23, AIC = 133.23). SNP2 and SNP3 significantly influenced the behavioral states, as well as genes *yellow-d2*, *BicC*, *Pif1*, *Exo84*, *KaiR1d*, *RD*, and *RFC3*, but not gene *PlexA* (Table S18). In contrast, within-colony relatedness, CHCs, and the environmental variables were never associated with the start of aggression.

The SNPs and DEGs contributed significantly to the behavioral associations, but the two SNPs and gene *BicC* contributed the most. Overall, the odds ratios of the SNPs and DEGs being associated with the behavioral states ranged from −4.13 to 4.90 (Table S18; Figure 3D). The highest log_n_-values were found for SNP2, which were 4.90 times higher for *reacted aggressively* and 3.80 times higher for *reacted peacefully* compared with *started aggression*. The next highest values were in *BicC*, which were 0.6 times higher for *reacted aggressively* and *reacted peacefully* compared with *started aggression*. The odds ratios that the DEGs *Exo84*, *yellow-d2*, *KaiR1d*, *Pif1*, *Rdl*, and *RFC3* were associated with the behavioral states were approximately 0.01–0.4 times higher for *reacted aggressively* and *reacted peacefully* compared with *started aggression*. For SNP3, values were −4.1 and −3.8 times lower for *reacted aggressively* and *reacted peacefully* compared with *started aggression*. The calculated pseudo-R^2^ value “Nagelkerke” was 0.75. In the pairwise comparison of the behavioral states (“emmeans”), the mean of *started aggression* was lower than the means of *reacted aggressively* and *reacted peacefully* (means + confidence intervals *started aggression* 0.07 + 0.02–0.12, *reacted aggressively* 0.38 + 0.26–0.50, *reacted peacefully*, 0.55 + 0.42–0.69; contrasts estimate *started aggression* vs. *reacted aggressively*: −0.31; df = 22, t-ratio = −5.04, *p* value = 0.001; estimate *started aggression* vs. *reacted peacefully*: −0.49; df = 22, t-ratio = −6.37, *p* value < 0.001), but not for *reacted aggressively* vs*. reacted peacefully* (estimate: −0.18; df = 22, t-ratio = −1.521, *p* value = 0.303). Additionally, we conducted log likelihood ratio tests to evaluate the significance of each focal variables by comparing a full model and a model that lacked the focal variable. Each focal variable contributed significantly to the respective model (Table S18; all models converged).

### Gene-enrichment analyses and gene-function predictions of the identified SNPs and DEGs

We used the identified SNP and DEGs, namely SNP3, *yellow-d2*, *BicC*, *Pif1*, *Exo84*, *KaiR1d*, *RD*, *RFC3*, and *PlexA*, in a gene-enrichment analysis and found that the identified are linked to depression restoration, synaptic and neurological functions, aggression, as well as plasticity. SNP2 was excluded as it was unknown. We conducted the gene-enrichment analysis in g:Profiler (using only annotated genes and FDR adjusted with *p*values < 0.05; (N_workers_=109) to test whether they were enriched for biological processes, molecular function, and/or cellular components. We used the fruit fly *Drosophila melanogaster* as a background gene set and conducted an unordered query to analyze whether certain biological pathways or gene sets were overrepresented. To increase the sample size of the gene-enrichment search, we used all genes regardless of whether they were up or downregulated or from different comparisons (for details, see the section “gene-enrichment analyses with known genes” in the supplemental results). In total, six molecular functions, 12 biological processes, and eight cellular components were enriched (Table S19). The molecular functions can be broadly summarized into signal transduction and binding and enzymatic and catalytic functions. The biological processes can be summarized into neural signaling, ion transport dynamics, gene expression regulation, and DNA replication and elongation. The cellular components can be summarized into replication functions, vesicle transport, neuronal functions, and ion channel functions. Overall, we combined them into two categories, namely neurological and synaptic functions as well as DNA replication, repair, and genome stability functions (Figure 3E). The former included the genes *BicC*, *Exo84*, *gd* (i.e., SNP3 in the gene *gd*), *KaiR1d*, *PlexA*, *Rdl*, and *yellow-d2*, while the latter included *Pif1 and RFC3*.

Using a gene-prediction analysis, we also identified neurological and synaptic functions across the SNPs and DEGs. In detail, we used GeneMania^39^ (FDR < 0.05; including gene *PlexA* because excluding *PlexA* only yielded non-significant results), which predicts gene function and searches for similar functional and related genes based on the initial gene list to find gene pathways or interactions. We included the same SNPs and DEGs in two queries, once with and once without gene *gd* as it contains an SNP. In the query with *gd*, we detected five biological processes and two molecular functions (Table S20). The biological processes can be summarized as regulation during cell division and the molecular functions as membrane transport functions. In the query without *gd*, we detected six biological processes and four molecular functions (Table S20). The biological processes can be summarized as neuronal signaling and regulation during cell division and the molecular functions as membrane transport functions, possibly linked to synaptic activity and signaling.

## Discussion

Almost all animals display aggressive behavior, but our understanding of the underlying mechanisms that promote the start of aggression is limited. Here, we integrated, for the first time, chemical, microbiome, genomic, transcriptomic, and environmental analyses and assessed whether these traits promote the start of aggression and reactions to it in the ant *Tetramorium alpestre*. We tested workers that displayed either of three behavioral states, namely *started aggression*, *reacted aggressively*, and *reacted peacefully*, identified in the aggression assays. Using the microbiome data, we discovered nine OTUs across four bacterial genera, *Bacteroides*, *Lactobacillus*, *Prevotella*, and *Pseudomonas*, that were associated with the behavioral states. We also identified two genes with a SNP each that were associated with the start of aggression (whole-genome data; GEMMA analysis), namely one unknown gene (SNP2) and the gene *gastrulation-defective* (SNP3). We also found significantly upregulated (*N*=30) and downregulated genes (*N*=28; FDR corrected for multiple testing) when comparing the state *started aggression* vs. each state *reacted aggressively* or *reacted peacefully*. Finally, we integrated these SNPs, DEGs, as well as additionally collected colony and environmental variables (e.g., within-colony relatedness, CHCs, site-specific nitrogen and temperature values) in a multinomial logistic regression (multiple data layers jointly). We found that SNP2 and SNP3 (in the gene *gd*) as well as the DEGs *BicC*, *Exo84*, *KaiR1d*, *Pif1*, *PlexA*, *Rdl*, *RFC3*, and *yellow-d2* are associated with the behavioral states, while CHC and colony and environmental variables were not.

### CHC compounds represent population structure but are not associated with the behavioral states

Using the CHC compounds, genetic differentiation, and relatedness values, we corroborate the colony and population structure expected at the onset of this study. The PCA of the CHCs used 63 compounds, which is slightly more than found in a recent study on this species (N_CHCs_ = 50).^9^ We expected the single-queened colonies to be separated from each other and the multiple-queened colonies to be mixed among colonies due to lower and higher relatedness, respectively. The combined analyses of the PCA of the CHC compounds, pairwise CHC analysis between populations, the CHC hierarchical cluster analysis, the genetic differentiation (pairwise F_ST_ values of the genomic data), and relatedness values corroborated this expectation. In detail, workers of the colonies of population SQ-N are related to each other and likely have only one queen. This also explains the narrow distribution of the CHCs in the PCA, as CHC bouquets are genetically determined and environmentally tuned.^18^ Workers of the colonies of population SQ-A are not or little related and also likely have one queen. As a result, the distribution of the CHC bouquet in the PCA is wider. Workers from colonies of population MQ-N have a higher relatedness scattered across colonies. This indicates that they likely have multiple, possibly unrelated queens. Workers from different queens within the same colony have very different CHCs leading to a broader variety in PCA of the CHCs.

While the microbiome, SNPs, and DEGs were associated with the behavioral states (discussed in the next three sections), the CHC bouquet was not. This is interesting because CHC compounds differed significantly between the three populations tested. Differences in the CHC compounds can cause aggressive behavior in ants^19^ but not necessarily in every ant species.^40^ Here and in a previous study using this species,^9^ we did not find any association between CHC differences and the behavior. This could be due to our study design: due to the small size of these animals, we were only able to use workers either for CHC extractions or for aggression assays (and subsequent genomic and transcriptomic analyses). By coincidence, the CHC bouquets of all fighting workers may have been more dissimilar from each other than the ones of the workers used for CHC analyses. Apart from this appearing unlikely as a pattern throughout, also a previous study on this ant^9^ and other ant species^19^^,^^40^ used different ants for CHC extractions and for aggression tests. They found a correlation^19^ or not^40^ suggesting that such a correlation could have been found if CHCs were important drivers of aggression in this ant. In contrast, this is the second study using this species indicating that CHC differences are not important for aggression in this species. Even though CHCs do not seem to elicit aggression in this species, we speculate that it still uses CHCs for nestmate recognition, but workers simply remain peaceful toward non-nestmates with different CHC bouquets.

### Gut bacteria are associated with the behavioral states

We found nine gut bacteria across four genera, *Bacteroides*, *Lactobacillus*, *Prevotella*, and *Pseudomonas*, that were associated with the behavioral states. The genus *Bacteroides* is linked with the behavioral states. We found a higher frequency of one *Bacteroides* sp. (OTU 1598) in ants that were aggressive (*started aggression* and *reacted aggressively*) than in ants that *reacted peacefully* as well as a higher frequency of one *Bacteroides* sp. (OTU 22324) in ants that *reacted aggressively* and *reacted peacefully* than in ants that *started aggression*. Tillisch et al.^20^ found that women with a higher *Bacteroides* abundance had more dense white brain matter tracts, indicating altered sensory processing. This may indicate that *Bacteroides* bacteria affect how ants perceive other ants and react accordingly. Additionally, Lin et al.^41^ and Strandwitz et al.^42^ found that a reduced abundance of *Bacteroides* bacteria in the gut is possibly linked with depression in humans. In turn, this may indicate that ants with higher *Bacteroides* counts were positively stimulated and thus more proactive and reactive.

We found a lower abundance of *Lactobacillus mucosae* (OTU 813) in ants that *reacted peacefully*, but a higher abundance of *Lactobacillus* sp. (OTU 21141), compared with ants that *started aggression* or *reacted aggressively*. Recent studies also found links between *Lactobacillus* and nestmate recognition in honeybees^43^ as well as behavioral changes in dogs,^12^ *Drosophila* flies,^11^ and ants.^13^ However, the results are partially contradictory: for example, one study found a higher *Lactobacillus* abundance in phobic dogs,^12^ namely *Lactobacillus plantarum*, which has known psychobiotic properties.^44^ However, another study on dogs and *D. melanogaster* males found that the genus *Lactobacillus* was more frequently present in aggressive dogs and *D. melanogaster* males.^11^ While the underlying mechanisms remain unclear, a link between *Lactobacillus* and behavioral changes seems to appear.

Also the bacteria *Prevotella* can affect behavioral states. We detected a higher frequency of three *Prevotella* spp. (OTU 377, 1887, 20448) in ants that *started aggression* than ants that *reacted aggressively* or *reacted peacefully* as well as in *reacted peacefully* than in *reacted aggressively*. A study in humans found that women with a higher abundance of *Prevotella* gut bacteria displayed higher negative affect when shown images with a negative emotional content, which was associated with both functional and structural differences in the hippocampus.^20^ Speculatively, these associations of *Prevotella* with aggression may indicate an evolutionarily conserved pathway of these gut bacteria with negative stimuli, in humans and ants, and possibly other animals.

Lastly, also the genus *Pseudomonas* is connected with behavioral states. We found two *Pseudomonas* spp. (OTUs 366, 2442) that had a higher frequency in ants that *started aggression*. To our best knowledge, no study has so far linked *Pseudomonas* to behaviors such as aggression. However, other studies have linked *Pseudomonas* strains with, for example, a potential insecticide resistance^45^ or metabolizing insecticides.^46^ Notably, insecticides often affect neuronal or synaptic functions. It thus may be that *Pseudomonas* is connected to behavioral changes via such a metabolic pathway.

While additional bacteria such as *Acetobacter*, *Enterococcus*, *Fusobacterium*, and *Megamonas* have been found to affect behavior in humans,^20^^,^^36^ dogs,^12^ *Drosophila,*^11^ and ants,^13^ we did not find any association with these bacteria here. We acknowledge that these or other gut bacteria may have been detected by using an interaction model or increasing the sample size of the gut microbiome analyses (e.g., by including the MQ-N population, which was unfortunately missing due to lacking samples). Nevertheless, we already detected bacterial taxa multiple times that were linked to the behaviors with the analysis conducted. Future studies should include interaction models and increase the sample size to further corroborate these results.

Our gut microbiome results are in line with other research indicating that there is accumulating evidence of microbiome effects on the recognition and behavior of animals such as humans,^20^^,^^22^^,^^41^^,^^42^ dogs,^12^^,^^21^ *Drosophila*,^11^ cockroaches, locusts, and termites^44^ (and references therein), as well as ants.^13^ For example, studies also suggests that the microbiome affects the behavior via the gut-brain axis^12^^,^^47^: the authors suggest the gut microbiome “communicates” with the central nervous system in various parallel ways such as the vagus nerve, signaling mechanisms, and the production of neuroactive chemicals (e.g., serotonin, gamma-amino butyric acid “GABA”).^12^^,^^47^ In turn, the central nervous system also communicates with the gut microbiome,^12^ creating a feedback loop. While an overall association seems to emerge, the exact effects of specific bacteria on their hosts remain to be determined. Thus, further studies are needed to assess such bacteria-host interactions.

### SNPs and DEGs are also associated with behavioral changes

We found one SNP each in two genes as well as six DEGs associated with behavioral, neurological, and synaptic functions that may explain the observed behavioral states. SNP2 is at a site of an unknown gene and will not be discussed further, while SNP3 is at a site in the gene *gd (gastrulation-defective).* For this SNP in the gene *gd*, more ants that *started aggression* were heterozygous at the SNP site. Although we found no direct link between the gene *gd* and aggression, we speculate that there may be an indirect link. The activation of *gd* leads to the activation of the *Toll* pathway. The *Toll* pathway is conserved and involved in the development of the dorsal-ventral embryonic axis,^48^ but it also promotes the expression of the *transcription factor nuclear factor kappa B*, which has functional roles in neuroprotection and synaptic plasticity.^24^ Gene *gd* may thus be associated with brain synaptic activity and thus possibly with the start of aggression.

We further found six downregulated genes associated with depression restoration, synaptic and neurological functions, aggression, as well as plasticity. These genes, *BicC*, *Exo84*, *KaiR1d*, *Rdl*, *yellow-d2*, and *PlexA*, are downregulated in workers that *started aggression* (*PlexA* was kept here as it was driving significant results in the GeneMania analysis). In more detail, *BicC* is associated with depression restoration,^49^ *Exo84* with neurite differentiation,^50^ *KaiR1d* with baseline synaptic transmission,^51^ *Rdl* with neurotransmission and olfactory learning,^52^ *yellow-d2* with dopamine receptor signaling,^53^ and *PlexA* as a receptor fir semaphorsin^54^ (for more details on each gene, see the section “six downregulated genes linked to synaptic functions” in the supplemental discussion). The results indicate these SNPs and downregulated genes could be linked to behavioral states in this ant species. Specifically, their associations with neurological and synaptic functions could indicate a potential direct link between them and the start of aggression and reactions to it. Further, they may affect the behavior in this ant in a concerted way.

We also found two downregulated genes associated with DNA repair and replication. These genes, *Pif1* and *RFC3*, are not directly but indirectly associated with behavior. For example, Gidron et al.^55^ found that under specific conditions and repeated exposure to stressful situations, reactive oxygen species increased and can yield to DNA damage in animals. It may be that ants that *reacted* to aggression were more sensible to stressful situations (e.g., sampling and laboratory maintenance) leading to an increase in oxidative stress scavenging mechanisms, which can reduce indices of oxidative stress.^56^ In turn, this may explain the upregulation of such genes in these ants compared with ants that started aggression. However, further studies need to shed light on such potential associations.

Aggression and its possible positive effects can be adaptive.^1^^,^^4^ This is especially true if starting aggression leads to increased fitness.^1^ The start of aggression has been loosely associated with individual effects, such as changes in the gut microbiome,^11^^,^^12^^,^^13^^,^^20^^,^^21^^,^^22^ SNPs in genes^23^^,^^24^ or DEGs.^17^ In this study, we integrated—for the first time to our best knowledge—gut microbiome data with chemical, genomics, transcriptomics, environmental, and behavioral assays, using the ant *T. alpestre*. We identified nine gut bacteria, two mutations, and eight DEGs that are associated with the three behavioral states *started aggression*, *reacted aggressively*, and *reacted peacefully.* In contrast, chemical and environmental factors were not associated with the behavioral states. The nine gut bacteria found are known to influence aggression and other behaviors in several organisms, for example, via hormone signaling.^11^^,^^21^^,^^22^^,^^43^ The identified SNPs and DEGs were, among others, associated with neurological and synaptic functions. Based on these results, we speculate that these three traits can contribute the start of aggression, possibly in a synergistic mechanism.

### Limitations of the study

We acknowledge the possibility that the identified bacteria, SNPs, and/or DEGs may be false positives and affected by population structure or within-colony relatedness. However, we argue that this is unlikely because these bacteria, mutations, and DEGs were identified by using independent datasets, applying corrections for multiple comparisons to minimize retrieving false positives, and combining the datasets in a joint analysis (i.e., multinomial logistic regression). Since we also observed the same behaviors across different colony types and relatedness levels, we suspect that population structure and relatedness only negligibly affect the behavioral results. However, we acknowledge that neither population structure nor relatedness were directly included in the models. We also checked the robustness of the results by dropping focal variables using log likelihood ratio tests. We thus argue that the results of these three independent datasets represent three distinct lines of evidence suggesting that similar underlying mechanisms can contribute to the start of aggression or the reaction to it, for example via hormone and synaptic signaling. The observed behavioral states may thus be influenced by these factors in a concerted way. At the same time, we stress that our results are correlative but not causal. Additionally, also other factors, such as CHC compounds of the workers with different behavioral states or epigenetic changes (e.g., DNA methylation, histone modifications), which were not tested here, may contribute to the start of aggression and reactions to it, and should be tested in the future. Future studies should thus also test whether the identified gut bacteria and genes are functionally relevant. This could be tested by conducting aggression tests with ants that have been fed with these bacteria or with ants in which these known genes are knocked out, impaired, or over-expressed. It would further be interesting to ascertain whether the same gut bacteria, genes, or gene homologs are important for the aggressive or peaceful behavior in other (social) insects as well.

## Resource availability

### Lead contact

Requests for further information and resources should be directed to and will be fulfilled by the lead contact, Patrick Krapf (p.krapf@uva.nl).

### Materials availability

This study did not generate new unique reagents.

### Data and code availability

• DNA sequencing data have been deposited at NCBI project PRJNA1447136: SAMN56895669-SAMN56895776. RNA sequencing data have been deposited at NCBI project PRJNA1444574: SAMN56761122-SAMN56761203. Microbiome sequencing data have been deposited at ENA project PRJEB56593: ERS13586486-ERS13586550. All are publicly available.
• The genomic and transcriptomic VCF files, the GTF file, and code to analyze genomic, transcriptomic, and microbiome data, as well as data and R-script to re-analyse the data have been deposited at Zenodo under https://doi.org/10.5281/zenodo.19279701, and are publicly available.
• Any additional information required to reanalyze the data reported in this paper is available from the lead contact upon request.

## Acknowledgments

We thank Philipp Andesner and Elisabeth Zangerl for help during lab work and Marlene Haider and Markus Möst for helpful discussions during statistical analysis. We thank the government of Carinthia for issuing a sampling permit for the protected area “Mussen”. The LUCAS topsoil dataset used was made available by the European Commission through the European Soil Data Center managed by the Joint Research Center (JRC; http://esdac.jrc.ec.europa.eu/). The computational results presented here have been achieved (in part) using the LEO HPC infrastructure of the University of Innsbruck and the MACH2 Interuniversity Shared Memory Supercomputer. This study was financially supported by the FWF (Austrian Science Fund) under Award number P 30861 awarded to F.M.S. and by the European Union’s Horizon Europe program under Marie Skłodowska-Curie Actions (MSCA)—Postdoctoral fellowship grant agreement no. 101204375 awarded to P.K. The maps were created using Stadia Maps (https://www.stadiamaps.com), Stamen design (https://www.stamen.com), and OpenStreetMap (https://wwwp.openstreetmap.org/copyright). We also thank two anonymous reviewers for helpful comments on earlier versions of the manuscript.

## Author contributions

Conceptualization, F.C., M.A., W.A., A.S.M., F.M.S., and B.C.S.-S.; methodology, P.K., F.C., M.S., T.K., M.A., W.A., A.S.M., F.M.S., B.C.S.-S.; software, P.K. and T.K.; validation, P.K. and M.S.; formal analysis, P.K., M.S., G.P.A., and T.K.; investigation, P.K., M.S., G.P.A., T.K., and M.A.; resources, P.K., G.P.A., M.A., F.M.S., and B.C.S.-S.; data curation: P.K. and M.A.; writing – original draft, P.K.; writing – review and editing, P.K., F.C., M.S., G.P.A., T.K., M.A., W.A., A.S.M., F.M.S., and B.C.S.-S.; visualization, P.K., M.S., and T.K.; supervision, F.M.S. and B.C.S.-S.; project administration, F.M.S. and B.C.S.-S.; funding acquisition, F.M.S. and B.C.S.-S.

## Declaration of interests

The authors declare no competing interests.

## Declaration of generative AI and AI-assisted technologies in the writing process

During the preparation of this work, the lead author used ChatGPT in order to annotate parts of the R code for data analysis. After using this tool, the author reviewed and edited the content as needed and takes full responsibility for the content of the publication.

## STAR★Methods

### Key resources table

**Table:** 

REAGENT or RESOURCE	SOURCE	IDENTIFIER
**Biological samples**		

*Tetramorium alpestre*	Personal collection	323550

**Chemicals, peptides, and recombinant proteins**		

Hexane	Merck	Product number 139386
Ethyl acetate	Merck	Product number 34858
96% ethanol	Merck	Product number E7023
Paraffine oil	Merck	Product number 107174
Fluon	De Monchy International BV	GP1
n-pentane	Merck	Product number 1071771000
DNA extraction for whole-genome sequencing	Qiagen	QiAmp Micro DNA Kit
DNA extraction for microsatellite genotyping	Sigma	Sigma GenElute extraction kit
RNA extraction for transcriptome sequencing	Macherey-Nagel	Nucleospin RNA Kit
16S rRNA for gut microbiome DNA from the abdome	Qiagen	QIAamp DNA Mini kit
DNA extraction for microsatellite genotyping for gut microbiome data	Qiagen	DNEasy Blood and Tissue Kit

**Critical commercial assays**		

Library prep and sequencing for DNA and RNA samples were outsourced to IGATech	http://igatechnology.com/	125-bp paired-end sequencing on HiSeq2500
Microsatellite genotyping	Comprehensive Cancer Center DNA Sequencing & Genotyping Facility, University of Chicago, USA	ABI3730XL genetic analyser (Applied Biosystems, Foster City, USA)
16S RNA sequencing	Novogene	N/A

**Deposited data**		

All trimmed DNA sequence data generated in this study	NCBI	NCBI: PRJNA1447136
All trimmed RNA sequence data generated in this study	NCBI	NCBI: PRJNA1444574
The analyzed VCF files for the DNA and RNA-seq data and the GTF file as well as related scripts	Zenodo	https://zenodo.org/records/19279701
All microbiome data	ENA	ENA: PRJEB56593

**Oligonucleotides**		

The universal primer pair for gut microbiome analyses 515F (5′-GTGCCAGCMGCCGCGGTAA-3′) and 806R (5′-GGACTACHVGGGTWTCTAAT-3′) was used to target the V4 region of the 16S rRNA gene using a 2 × 250 bp approach	N/A	N/A

**Software and algorithms**		

Agilent ChemStation software (Agilent, Waldbronn, Germany).	Agilent	https://www.agilent.com/en/product/software-informatics/analytical-software-suite/chromatography-data-systems/openlab-chemstation
NIST database	National Institute of Standards and Technology (USA)	https://webbook.nist.gov/chemistry/
Chemical database of the Institute of Evolutionary Ecology and Conservation Genomics at the University of Ulm	University of Ulm	See ref.^57^^,^^58^
R version 4.3.0	R Core Team	https://www.R-project.org/
prcomp	ggfortify package^59^	N/A
ggfortify	ggfortify package^59^	N/A
adonis2	vegan package^34^	N/A
pairwise.adonis	pairwiseAdonis package^35^	https://github.com/pmartinezarbizu/pairwiseAdonis
hierarchical cluster analysis using agnes	cluster package^60^	N/A
hierarchical cluster analysis using Ward’s minimum variance method	cluster package^60^	N/A
prcomp	R Core Team	N/A
FastQC	FastQC	https://www.bioinformatics.babraham.ac.uk/projects/fastqc/
MultiQC	MultiQC	https://seqera.io/multiqc/
bbduk, bbtools, bbmap, callvariants	bbtools	https://sourceforge.net/projects/bbmap/
Relatedness calculation - Manichaikul et al. (2010)^33^	VCFtools^61^	N/A
Genome-wide association study	GEMMA^62^	N/A
Manhattan plot	qqman package^63^	N/A
Gene expression analyses	DESeq2 package^64^	N/A
pheatmap	pheatmap package^65^	N/A
gene enrichment analysis in g:Profiler	g:Profiler	https://biit.cs.ut.ee/gprofiler/gost
Venn diagram, ggvenn	ggvenn package^66^	N/A
flash v.1.2.7^67^ for merging files	N/A	N/A
Qiime v.1.7.0 for quality filtering	Qiime v.1.7.0	https://qiime.org/1.7.0/
SILVA v.138 as a reference database and to detect chimeric sequences	SILVA v.138	https://www.arb-silva.de/documentation/release-138/
phyloseq object	phyloseq package^68^	N/A
Principal Coordinate Analyses (PCoA) using ampvis2	ampvis2 package^69^	N/A
pairwise comparisons using “emmeans”	NA	N/A
anova function for model comparison	basic stats package	N/A
Akaike Information Criterion for small sample sizes (AICc) for using the “aictab” function	AICcmodavg package^70^	N/A
likelihood ratio test “lrtest” to test variable significance	lmtest package^71^	N/A
pairwise comparison of generalised linear models using emmeans	emmeans package^37^	N/A
Microsatellites were genotyped using GeneMarker V.3.0.1	GeneMarker	SoftGenetics, State College, PA, USA
GeneClass2 for individual allocation to colonies	GeneClass2	GeneClass2

**Other**		

WordlClim dataset^38^	WordlClim dataset^38^	https://worldclim.org/
European LUCAS topsoil dataset^72^	European LUCAS topsoil dataset^72^	https://esdac.jrc.ec.europa.eu/projects/lucas

### Experimental model and study participant details

#### Experimental model: Animals

All ant samples were collected by the authors of this study. We used female workers of the ant *Tetramorium alpestre* for this study. Males are short-lived (a few weeks only), and it was not possible to use males. Further, workers are more important as they conduct all the work in a nest such as taking care of the brood, maintaining the nest, foraging, etc. We thus chose to use workers. We collected roughly 500 workers from each mound and selected workers randomly from the nest. During the aggression assays, we only used active ants, which are likely foragers and thus have a similar age. However, it was not possible to determine the exact age of the workers used, which may have confounded the results. Nevertheless, we argue that by using active workers, these differences are negligible.

This study used ants from a non-threatened ant species. No license or permit was required to conduct assays with invertebrates including ants (ethics statement). Nevertheless, we followed the highest ethical standards and international law for ants collected and kept in the laboratory. We also followed two of the 3Rs Principle: i) We reduced the number of workers collected to a minimum (Reduction principle) and iii) we created the best accommodation possible for ant workers (Refinement principle). Ant colony fragments were collected in the field and maintained in the laboratory with care. Ants were provided with suitable nesting possibilities, and *ad libitum* food and water. After the experiments, colony fragments were kept in the laboratory and reared until their natural death.

### Method details

#### Fieldwork and colony maintenance

Between July 18^th^ and 25^th^ 2018, 500 workers were sampled from three colonies each in three populations (N_colonies_=9, Table S1). The populations were selected based on preliminary behavioural data (not shown): One population was located in South Tyrol, Italy, and comprised single-queened and aggressive colonies (“SQ-A”), one in Tyrol, Austria, comprising single-queened and non-aggressive colonies (“SQ-N”), and one in Carinthia, Austria, comprising multiple-queened and non-aggressive colonies (“MQ-N”; potentially supercolonial population). Of these 500 workers, 200 were immediately snap-frozen in the field using a dry shipper (CY50915D, Thermo-Fisher Scientific Inc., MA, USA) for CHC and molecular analyses. The remaining workers were transported alive to a laboratory at the University of Innsbruck and transferred to polypropylene boxes (10.5 × 10.5 cm; as of now “colony”) awaiting behavioural assays. These workers presumably included all polyethism stages.^31^ To prevent workers from escaping, the walls of the boxes were Fluon-coated (GP1, De Monchy International BV, Rotterdam, Netherlands). Each box was equipped with soft tissue as a hiding place, two conical Eppendorf tubes filled with water or with diluted honey water and each plunged with cotton as a drinking aid, and a frozen *Drosophila hydei* fruit fly. The water, honey water, and fruit fly were refilled twice per week and present at all time *ad libitum*. The boxes were placed in a climate cabinet (MIR-254, Panasonic, Etten Leur, Netherlands) with constant dark conditions, a humidity of 50-70%, and at constant 18 °C. Constant 18 °C was selected to acclimatise workers that originated from slightly different elevations to a similar temperature. Before the various assays, the colonies were kept in the climate cabinets for two weeks. Pairwise geographic distances between populations SQ-N:MQ-N (Kuehtai – Mussen; Figure 1A) were 140 km, between SQ-A:MQ-N (Penser Joch – Mussen) 125 km, and between SQ-A:SQ-N (Penser Joch - Kuehtai) 40 km calculated using an online tool (https://www.ibm.franken.de/gps03.html).

#### Recognition assays

Between August 13^th^ and 20^th^ 2018, we conducted recognition assays to test if workers recognise and prefer their own colony odour over an alien colony or a control odour following Steiner et al.^26^ For these assays, we extracted cuticular hydrocarbons (CHCs) from workers of each colony separately using three different extraction solvents sequentially, starting in 100 μl hexane, then 100 μl ethyl acetate, and lastly 100 μl 96% ethanol (all three Merck, MA, USA).^26^ For the extraction, we transferred the workers into 1.1-ml conic glass vials (CZT, Kriftel, Germany). Workers remained in each extraction solvent for 90 s before being transferred to the next. The three solvents should extract as many CHCs as possible. We then transferred the workers to 96% ethanol, mixed the three solvents, and stored them at -20 °C until further use. To account for body size differences, we used 15 workers for each colony from populations SQ-A and SQ-N and 22 workers for each colony from population MQ-N, which had smaller workers. In total, we generated nine solvents (one for each colony) to test if workers prefer their own, alien, or control (a mixture of the three extraction solvents without CHCs) odour. To do this, we created small filter paper disks (2 cm diameter; 75 g/qm, Altmann Analytik, München, DE) with three 120-degree sectors (own, alien, and control sector). Onto the sector “own”, we applied 1 μl of the extract of the colony to be tested, onto the sector “alien”, 1 μl of the extract of a different colony from the same population, and onto the sector “control”, 1 μl of the mixture of extraction solvents without CHCs. We transferred the solvents onto the paper disks using a 20-μl syringe (Hamilton, NV, USA). After transferring the solvents onto the papers, the solvents were left to evaporate for three minutes before we transferred the paper disks to the bottom of small glass vials (2 cm Ø). The bottom of each glass vial was covered with 1 μl paraffin oil (Merck, MA, USA) as a keeper substance.^26^ The walls of the glass vials were Fluon-coated to prevent workers from escaping. After evaporation, we transferred individual workers to the glass vials, which were covered with tin cans to simulate dark conditions. Workers were allowed to acclimatise for 15 minutes, after which we lifted the can for five seconds and noted the sector of the paper disk on which the worker was sitting. After each observation, we turned the vial 120 degrees and lightly tapped it thus forcing the worker to move. In each assay, we tested all colonies in a randomised order. Both conductors and evaluators were blind to the origin of colonies. We conducted the assays in an air-conditioned room with constant 18 °C resembling the temperature in the climate cabinet. In one run, 36 workers were tested (four from each colony), and 15 runs were conducted. This procedure was replicated three times over three days resulting in 1,620 observations, which were analysed together using a multinomial Goodness-of-Fit test to test if workers recognise and prefer their own colony odour, an alien colony odour, or a control odour.

#### Extraction and analysis of cuticular hydrocarbons (CHCs)

We extracted CHCs from five workers per colony following Krapf et al.^9^ For the extraction, we transferred five workers, which had been immediately frozen after sampling, to 1.1-ml conic glass vials (CZT, Kriftel, Germany) and immediately added 100 μl n-pentane (Merck, MA, USA) using a 100-μl syringe (Hamilton, NV, USA). The CHCs were extracted for three minutes while the glass vials were being shaken at 450 rpm. After the extraction, we removed the workers from the vials and transferred them to Eppendorf tubes filled with 96% ethanol. The vials containing the CHC extracts were sealed until their analysis. For the analysis, a 7890 B Series gas chromatograph (Agilent, Waldbronn, Germany) equipped with a flame ionization detector (FID), a nonpolar DB-5 column (30m×0.25 mm inner diameter, J&W, Waldbronn, Germany), and hydrogen (2ml/min constant flow) as carrier gas was used. One μl of a sample was injected splitless at an initial oven temperature of 50 °C. After 1 min, the splitting valve was opened and the temperature gradually increased by 10 °C/min until it reached a final temperature of 310 °C, which was kept constant for 50 min. To ensure the consistency of the analyses, GC runs were performed regularly with a synthetic alkane standard mixture. Structure elucidation of individual compounds was performed with an HP (Hewlett Packard) 6890 Series gas chromatograph connected to a mass selective detector (GC–MS; Quadrupole 5972, Agilent, Waldbronn, Germany). Helium was used as carrier gas (1.5 ml/min constant flow). The temperature program was the same as described above. The absolute and relative amounts of these compounds were determined by using Agilent ChemStation software (Agilent, Waldbronn, Germany). Structure assignments were carried out by comparison of mass spectra and retention times of natural products with corresponding data from synthetic reference samples using the NIST database and a database of the Institute of Evolutionary Ecology and Conservation Genomics at the University of Ulm, following previous work.^57^^,^^58^ Peak identities across different runs were confirmed by GC-MS.

To estimate relative proportions for further downstream analyses, we only used CHCs that were found in all samples. Further, we divided the absolute amounts of individual compounds by the sum of the absolute amounts of all compounds and multiplied by 100. With these CHC compounds, we created a PCA using the function “prcomp” (“ggfortify” package^59^) to check if colonies and/or populations form distinct clusters. Further, we first compared the CHC compounds between the three populations using a Permutational Analysis of Variance using the adonis2 function (PERMANOVA; vegan package^34^ with 4999 permutations and “Bray” distance) and, second, pairwise compared them using the pairwise.adonis function (pairwiseAdonis,^35^ https://github.com/pmartinezarbizu/pairwiseAdonis). Lastly, we conducted a hierarchical cluster analysis with the CHC data using the function “agnes” and Ward’s minimum variance method (“cluster” package^60^). We used the values of the first PCA in the multinomial regression.

#### One-on-one aggression tests

We conducted one-on-one aggression tests within each population on July 23^rd^ 2018 to determine if the colonies displayed the expected behaviour (i.e., aggressive and non-aggressive). We conducted standardised aggression tests^9^ in an air-conditioned room with constant 18 °C. For each aggression test (i.e., one encounter), we randomly selected naïve single workers from different colonies from the same population and transferred to a small glass vial (1.4 cm inner diameter) with Fluon-coated walls preventing workers from escaping. Only workers actively running outside in the arena were selected, which likely were foragers.^31^ We first added a worker from one colony, then started the video, and lastly added the second worker, which allowed us to track the movement of both workers and thus differentiate them in the subsequent video analysis. In the next encounter, we changed the order of workers introduced to prevent any effect of adding workers to the vial. We conducted five encounters for each colony combination to account for behavioural variation.^73^ Each encounter lasted 180 s and was filmed using high-definition cameras (Handycam HDR-XR 155; HDRPJ810E, Sony, Tokyo, Japan). As workers might have been agitated after being transferred to the vials, the first 10 seconds of each encounter were regarded as an acclimatisation time and were thus excluded from further analyses.^9^^,^^30^^,^^31^ The assay conductors were not blind to the colony’s origin.

We further conducted one-on-one aggression tests between populations between July 25^th^ and 28^th^ 2018 following the approach described above. Within 10 minutes after the end of the aggression test, we separated the workers if fighting, transferred them individually to 1.5 ml tubes, and snap-froze them using liquid nitrogen. This procedure ensured that no early genes were expressed, which can start after 15 minutes.^74^ At this point, the colony origin of the workers was unknown, but we later identified the colony identity using microsatellite analysis (see section below “microsatellite genotyping for reference workers”). Additionally, we conducted within-colony aggression tests on July 27^th^ 2018 to test if workers behaved peacefully, which was our expectation.

#### One-on-one aggression analysis and worker selection for sequencing

For an initial screening of the aggression test, we noted the behaviour of both workers every ten seconds as “aggressive” (*i.e.*, any kind of biting or fighting behaviour), “neutral” (*i.e.,* being physically distant from each other), or “peaceful” (*i.e.,* antennation, being in close proximity to each other) while conducting the aggression tests. Based on this initial screening, we selected 112 videos for a detailed analysis. From these videos, we examined the behaviour of each worker in slow-motion, and classified the behaviour of both workers second by second using the following scoring scale^75^: (−4) trophallaxis, (−3), allogrooming, (−2) antennation, (−1) being next to each other without contact, (0) ignoring, (1) avoiding, (2) mandible threatening, (3) fighting without gaster flexion, (4) fighting with gaster flexion, and (5) killing. The observer of the videos was blind to the origin of the colonies. Moreover, an aggression index *AI*^76^ was calculated as detailed in Krapf et al.^9^ For *AI*, the duration of each behaviour was summed up and multiplied by its respective behaviour score (-4 to +5). This value was divided by the total number of seconds with tactile interactions recorded. Lastly, the arithmetic mean of the five replicates was calculated.

Using this detailed analysis, we defined three behavioural states: workers that ‘*started aggression’* (*i.e.,* a worker that attacks another worker), workers that *‘reacted aggressively’* (*i.e.,* a worker that fights back by pulling or biting when it is attacked by another worker), or workers that *‘reacted peacefully*’ (*i.e.,* a worker that remains motionless or does not change its behaviour when being attacked by another worker). For the aggressive states (*started aggression*; *reacted aggressively*), we used workers that displayed a scoring value of 3 and higher to ensure that high aggression levels were used. We tested aggression levels using an Analysis of Variance and subsequent Tukey Honest Significant Post-Hoc Test (significant differences are represented with asterisks, ∗∗∗ = <0.001; ∗∗ = <0.01; ∗ = <0.05). Based on these three behavioural states, we selected 109 workers for whole-genome sequencing (*started aggression* = 43 workers, *reacted aggressively* = 35 workers, *reacted peacefully* = 31 workers; see Table S1 for population and colony details) and, of those, we selected 85 workers for transcriptomic analyses (*started aggression* = 31 workers, *reacted aggressively* = 29, *reacted peacefully* = 25). The additional 24 workers selected for whole-genome sequencing originated from the non-aggressive and polygynous population MQ-NS. They were used to account for multiple queens and reliably calculate within-colony relatedness and estimate queen numbers.

#### DNA- and RNA-extractions and whole-genome and whole-transcriptome sequencing

For whole-genome sequencing of samples, we cut off the mesosoma and abdomen from the head of each ant using sterile scalpels (Figure 1P). We used the mesosoma and abdomen for DNA extractions (N_samples_=109) and the head for RNA extractions (N_samples_=85). We extracted DNA using the QiAmp Micro DNA Kit (Qiagen, Hilden, Germany). For this, we transferred the mesosoma and abdomen of each worker to a sterile tube and submerged it into liquid nitrogen. We then ground the mesosoma and abdomen using disposable pestles. The extraction followed the manufacturer’s protocol except for the dilution, which was conducted twice, as follows: the first elution was done with 50 μl dH_2_0 for whole-genome sequencing and the second elution with 30 μl dH_2_0 for microsatellite genotyping to determine the colony affiliation (see section below “microsatellite genotyping to identify colony identity”).

We extracted RNA from the heads of 85 workers using the Nucleospin RNA Kit (Macherey-Nagel, Düren, Germany) following the manufacturer’s protocol. For this, we transferred the head of each worker to a sterile tube, submerged the tube into liquid nitrogen, and grinded the head using disposable pestles. The subsequent extraction followed the manufacturer’s protocol except for the dilution: RNA was eluted in 40 μl RNAse-free dH20 provided by the manufacturer.

We conducted all DNA- and RNA-extraction steps under sterile conditions in a laminar flow hood. DNA and RNA extracts were stored at -70 °C until being shipped for library preparation and whole-genome and -transcriptome sequencing outsourced to a commercial provider (IGATech, http://igatechnology.com/). Each worker was sequenced with 125-bp paired-end sequencing for both DNA- and RNA extractions on HiSeq2500.

#### Microsatellite genotyping to identify colony identity

We conducted microsatellite genotyping to assess the colony identity of workers used in aggression tests. First, we genotyped 12 reference workers from each colony (i.e., known colony identity) using eight microsatellite loci.^9^^,^^30^ For this, we extracted DNA using the Sigma GenElute extraction kit following the manufacturer’s protocol, except for eluting in 50 μl. PCR for genotyping was done in 5 μL reaction volume with 0.5 μL template DNA, 2 × Rotorgene Master Mix (Qiagen, Hilden, Germany), 0.01 μM M13 tailed locus-specific forward primer, 0.1 μM fluorescent-labelled M13 primer, 0.1 μM untailed specific reverse primer, and 1.79 μL dH_2_O on a UnoCycler 1200 (VWR, Radnor, USA). Cycling conditions were 94 °C for 5 min followed by 35 cycles at 94 °C for 30 s, 60 °C for 1 min, 72 °C for 45 s, and a final extension at 68 °C for 20 min. Fragment analysis was carried out on an ABI3730XL genetic analyser (Applied Biosystems, Foster City, USA) by a commercial provider (Comprehensive Cancer Center DNA Sequencing & Genotyping Facility, University of Chicago, USA). Microsatellites were genotyped using GeneMarker V.3.0.1 (SoftGenetics, State College, PA, USA).

Following the same procedure, we genotyped workers from the aggression tests and reliably assigned the colony identity before shipping the samples to the commercial provider IGA for sequencing. Based on the genotypes of known colony identities, we calculated the probability of colony affiliations using the software GeneClass2.^77^ GeneClass2 uses multilocus genotypes to select or exclude populations as origins of individuals. To find colony affiliations, we chose the Bayesian method by Rannala & Mountain (1997)^78^ as the computation criteria, and the assignment threshold of the scores was 0.05. Further, we calculated within-colony relatedness based on the genotypes following Queller and Goodnight algorithm^79^ and additionally, the number of queens following Pamilo (1991).^80^

#### Analysing whole-genome and whole-transcriptome sequences

For both DNA and RNA files, we conducted the same analysis approach. Initial quality control of raw reads was conducted using FastQC (https://www.bioinformatics.babraham.ac.uk/projects/fastqc/) and MultiQC (https://seqera.io/multiqc/). We trimmed adapters, duplicates, and contaminants using a “kraken” database and “bbduk” (“bbtools”, https://sourceforge.net/projects/bbmap/). We merged trimmed paired-end files into single files and mapped single files against the *Tetramorium alpestre* reference genome^81^ using “bbmap” (bbtools) by applying quality trimming on both sides. For mapping, we indexed the files and quality-trimmed them using “bbmap” (minid=0.9, k=13). We called single nucleotide polymorphisms (SNPs) using the “callvariants” function from “bbtools” using the default settings except for ploidy=2. For variant calling, we first called variants in an initial VCF file. Second, we calculated the true equality and then recalibrated them using the initial VCF file. Third, we created an unfiltered VCF file. In this unfiltered VCF file, we identified 1,249,705 and 312,297 SNPs for whole-genome sequences and whole-transcriptome sequences, respectively. We further filtered this unfiltered VCF file using a minimum coverage of 128, a minimum number of sequences of 4 with the alternative allele, a minimum mapping quality of 50, and including linkage-disequilibrium (LD) pruning. After filtering, 184,145 and 69,191 SNPs were kept in the final whole-genome and whole-transcriptome VCF file, respectively.

Using the VCF file of the whole-genome data, we calculated the heterozygosity and Weir and Cockerham's F_ST_ and created an LD-pruned PCA using VCFtools.^61^ We further calculated the within-colony relatedness using the “relatedness” function in VCFtools^61^ using the method of Manichaikul et al.^33^ We then compared the within-colony relatedness from whole-genome data with the within-colony relatedness from microsatellite genotyping to assess concordance of values (Table S3).

### Quantification and statistical analysis

#### DNA and RNA data

##### Genome-wide mixed-model association (GEMMA) analysis using whole-genome sequences

We conducted a GEMMA^62^ analysis using whole-genome sequence data to determine if the behavioural states were associated with SNPs in the VCF. Before the analysis, we excluded duplications in the VCF to reduce the bias of emphasising duplications. We used this VCF file without duplications to create a bimbam file using a custom-made Python script. After calculating the bimbam file, we calculated a centred relatedness matrix using “gemma”, which was used in the subsequent GEMMA analysis. In the GEMMA analysis, a phenotype list detailing the behavioural states of workers, a bam list, and an LD-covariance file were used. GEMMA results were visualised using Manhattan plots created in R using the function “Manhattan” (“qqman” package^63^). We inspected genomic SNPs above the suggestive line by using them a PCA created with the function “prcomp” (“ggfortify” package^59^) to check whether alleles clustered together. For this PCA, we dummy-coded individuals that were homozygous for the reference allele of the respective genes as 0/0 and individuals that were heterozygous for the reference alleles as 0/1. We did not find any individual that was homozygous for the alternative allele. Further, we conducted a Pearson’s Chi-squared test for count data with simulated *p*-value and 2000 Monte Carlo replicates to calculate the *p*-values. The count data represented the number of counts of all individuals for being homozygous or heterozygous for the reference allele for the three behavioural states. The idea was to check if individuals that were homozygous or heterozygous for the reference allele were more or less frequently observed in one of the three behavioural states.

##### Differential gene expression and gene-enrichment analysis

Differential gene expression: The expression counts of each individual stemming from a newly created annotation (for details, see the section “tetramorium alpestre annotation” in the supplemental materials and methods) were merged using a customised R script. Using this merged data set, we analysed the expression counts of all individuals (“DESeq2” package^64^). For this, we created a DESeqDataSet object to compare the expression of the behavioural states in a pairwise manner. The three behavioural comparisons were: *started aggression* vs *reacted aggressively*, *started aggression* vs *reacted peacefully*, and *reacted aggressively* vs *reacted peacefully*. As a pre-filtering step, we only kept genes that had at least 10 counts across all samples, thus excluding genes with counts below 10. Next, we assessed the data quality of each sample using a pheatmap (“pheatmap” package^65^). Of the 85 samples, we excluded three due to low quality, yielding 82 samples for subsequent analysis. We conducted a differential gene expression analysis with these 82 samples based on the Negative Binomial (i.e., Gamma-Poisson) distribution and using the default settings. We created volcano plots for each behavioural comparison exported the results as table with a log fold change threshold of zero and using a of 0.05-Benjamini-Hochberg correction (“result” function; DESeq2 package; “false-discovery rate”, FDR). We created such result tables for all three comparisons and up-regulated as well as down-regulated genes separately and used them in subsequent analyses. Such tables included gene names, log_2_fold values, p-values, and FDR-corrected p-values for multiple testing. We further queried gene names in FlyBase (release FB2025_04) to obtain information on gene function. In subsequent gene-enrichment analyses and multinomial logistic regression analyses, we only used genes with a known (i.e., annotated) gene name.

Gene-enrichment analyses: For the three behavioural comparisons *started aggression* vs *reacted aggressively*, *started aggression* vs *reacted peacefully*, and *reacted aggressively* vs *reacted peacefully*, we conducted a gene enrichment analysis in g:Profiler (https://biit.cs.ut.ee/gprofiler/gost), a web server for functional gene-enrichment analysis. We only used known (i.e., annotated) genes with an FDR-adjusted p-value lower than 0.05 (Table S5). For each behavioural comparison, we conducted a query with an unordered list of genes based on the log_2_fold changes. We selected *Drosophila melanogaster* as the organism to match the query gene list. Further, we created Venn diagrams using the behavioural-comparison genes for the annotated and all genes in R using the function “ggvenn” (“ggvenn” package^66^). This analysis allowed checking whether the same genes are up- or down-regulated in several comparisons.

##### Microbiome DNA extraction and marker gene sequencing

To test whether the microbiome influenced the three behavioural states, we conducted 16S rRNA gene sequencing. Due to a limited availability of samples, we used 49 workers from two populations: Specifically, we selected four workers each from two colonies of the single-queened and aggressive population SQ-A (colonies SQ-A5 and SQ-A6) and from two of the single-queened and non-aggressive population SQ-N (colonies SQ-N1 and SQ-N6; SQ-N6 with five workers) and from each behavioural state. This resulted in using 16 workers that *started aggression*, 16 that *reacted aggressively*, and 17 that *reacted peacefully* (Table S1). To test if the microbiome changed during laboratory maintenance, we selected 16 additional workers (4 workers each from the colonies SQ-A5, SQ-A6, SQ-N1, and SQ-N6) as control. These workers were immediately frozen after fieldwork and did not experience any laboratory maintenance.

Before the extractions, we sterilised the surface of whole workers by transferring individual workers for 15 s into Eppendorf tubes filled with 100 μl 5% bleach and then for 15 s into Eppendorf tubes filled with 100 μl phosphate-buffered saline solution (PBS; 137 mM NaCl, 2.7 mM KCl, 10 mM Na2HPO4, and 1.8 mM KH2PO4).^82^

For the 16 control workers, we extracted DNA using the QIAamp DNA Mini kit (Qiagen, Hilden, Germany) and eluted twice each time with 30 μl of the elution buffer from the kit. For the remaining 49 workers, we dissected the heads from the mesosoma and gaster using a sterile scalpel. For microbiome analyses, we extracted DNA from the mesosoma and gaster using the QIAamp DNA Mini Kit. To determine colony affiliation using microsatellite genotyping (for details, see “microsatellite genotyping of reference workers” above), we extracted DNA of the head using the DNEasy Blood and Tissue Kit (Qiagen, Hilden, Germany). We extracted DNA following the manufacturer’s protocol except for the elution: DNA was eluted twice each time with 30 μl of the elution buffer from the kit. We conducted all steps before and during the extraction under sterile conditions in a laminar flow hood. High-quality DNA extracts were sent to Novogene (Cambridge, United Kingdom) for marker gene sequencing on a NovaSeq6000 machine (Illumina, San Diego, CA, United States). The universal primer pair for bacteria 515F (5’-GTGCCAGCMGCCGCGGTAA-3’) and 806R (5’-GGACTACHVGGGTWTCTAAT-3’) was used to target the V4 region of the 16S rRNA gene using a 2×250 bp approach.

#### Microbiome data

##### Analysis of 16S rRNA gene-sequencing data

We merged the raw reads into contigs using flash” v.1.2.7.^67^ We used Qiime v.1.7.0 (https://qiime.org/1.7.0/) for quality filtering following the standard operating procedures. We used SILVA v.138 (https://www.arb-silva.de/documentation/release-138/) as a reference database and to detect chimeric sequences by the UCHIME algorithm, which we removed from the data. Sequences were clustered into OTUs based on a ≥97% similarity threshold. We converted the raw data to a phyloseq object (“phyloseq” package^68^) and rarefied to the smallest sample size, after removing the sample Nu_ctrl_153a as an outlier. We conducted principal coordinate analyses (PCoA) based on populations and behavioural states and visualized the data (“ampvis2” package).^69^ In total, we found 22,215 OTUs after rarefaction. We further calculated the frequency of the four most frequent bacterial genera as well as for four additional bacteria genera, *Acetobacter, Enterococcus, Fusobacterium, Megamonas,* and the orders Rhizobiales and Entomoplasmatales. *Acetobacter,* and *Enterococcus* have been associated with aggression in *Drosophila melanogaster,*^11^ *Fusobacterium* and *Megamonas* have been associated with aggression and non-aggression in dogs,^12^^,^^21^ and Rhizobiales and Entomoplasmatales have been associated with aggression in leaf-cutting ants.^13^ Entomoplasmatales were not found in our data set.

Using the bacterial OTU genera mentioned above, we selected OTUs that had a frequency of at least 100 across the behavioural states (*N*=119), thus focusing on the most frequent OTUs and restricting the analysis to 119 OTUs. With these, we conducted a sliding-window approach with multinomial logistic regressions (function “multinom”, “nnet” package^83^). A multinomial regression allows using more than one categorical variable as response variables (here, the three behavioural states). In the sliding-window approach, we created individual models that tested 20 OTUs simultaneously in a multinomial regression. Briefly, the first model used OTUs 1 to 20, the second model OTUs 2 to 21, etc. To evaluate the model fit and calculate *p*-values and log-likelihood tests, we conducted the same methods as described in the section “combining SNPs, DEGs, CHCs, relatedness, and environmental variables counts in a multinomial regression”.

In each model (*N*=119), we used the behavioural state *started aggression* as the baseline. Manually checking 119 model fits and results was not efficient, so we created an R (version 4.3.0^84^) script to extract model fits and model *p*-values for the different OTUs. The script also counted how often OTUs were significantly influencing the behavioural states and thus allowed checking if the same OTUs influenced the behavioural states more or less frequently. Our rationale was that if one or a few OTUs are present in many or all models, then these OTUs likely have a higher impact on the behavioural states than OTUs with a low frequency. If, however, OTUs are only counted a few times, they have likely arisen due to chance and may represent artefacts. From these models, we extracted the significant OTUs and counted their frequency across the models.

Across these models, the most frequent OTUs (N_OTUs_=58 with a frequency ≥10) included the genera *Bacteroides* (relative percentage across the 58 models, 25%), *Lactobacillus* (9%)*, Prevotella* (43%)*, Pseudomonas* (17%), the order Rhizobiales (4%), and the genus *Fusobacterium* (1%). For these gut bacteria, we noted the OTU frequency in each behavioural state and the control. From the 58 OTUs, we excluded 40 OTUs (five because the frequency was significantly higher or lower than in the control, 16 OTUs because the count of the control was higher as the highest number of counts of one of the behaviours, six OTUs because the counts were evenly distributed across all behavioural states, five because the counts were less than 10 in one of the behavioural states, seven OTUs because the counts of the control was similar as the counts of the behavioural states, and one because the counts were not different between the control and the behavioural states) yielding 18 OTUs for further analyses, namely three *Bacteroides* spp., three *Lactobacillus* spp., nine *Prevotella* spp., three *Pseudomonas* spp., and one Rhizobiales sp.

With this set of 18 OTUs, we assessed whether the counts differed between the behavioural states. For this, we conducted a generalised linear model with these count data (response = count; explanatory variable = behavioural states; Poisson-distributed) and assessed the pairwise comparisons (“emmeans” package; Tukey corrected for multiple testing), which also revealed statistical values (∗∗∗ = <0.001; ∗∗ = <0.01; ∗ = <0.05 by generalised linear model and pairwise comparisons). Nine OTUs revealed significant results and were further discussed, while others were non-significant or revealed inconsistent results (*i.e.,* reacted aggressively higher than the other behavioural states). We could not analyse the microbiome data together with SNPs and DEGs because no samples for the population MQ-N were available for the microbiome analysis.

##### Environmental variables used in the multinomial regression analyses

For each colony, we estimated a standardised air temperature (TAS) as a rough measure of the colonies’ thermal niche.^85^ Following the logic of Seifert and Pannier,^86^ TAS was calculated for a sampling site as the mean air temperature of the period from May 1st to August 31st averaged over the years 1961 to 1990 of the nearest three meteorological stations (data provided by Klimaabteilung der Zentralanstalt für Meteorologie und Geodynamik (1996), Vienna, Austria). The data were corrected for an altitudinal decrease in temperature of 0.661 °C per 100 m according to the equation of Seifert and Pannier^86^:

(Equation 1) TAS=−0.694×LAT+0.078×LON−0.00661×ALT+52.20,

where TAS is the predicted standardised air temperature in °C, LAT and LON denote the geographical latitude and longitude in decimal format, respectively, and ALT is the altitude above sea level in metres.

From the WordlClim dataset,^38^ we downloaded environmental variables from the years 1970 to 2000 and extracted site-specific values using the “extract” function (“raster” package^87^). In particular, we selected data on mean annual precipitation, precipitation of the warmers quarter, mean annual temperature, and the maximum temperature of the warmest month both as temperature and precipitation affect the colonies’ environment and higher temperatures promote aggression in this species.^9^ Further, we retrieved soil nitrogen values for each site from the European LUCAS topsoil dataset.^72^ We used these variables in multinomial regression analyses (described in the next paragraph) to test if the environment is associated with the behavioural states. We recently found such an association in this ant, where higher temperature and nitrogen values were positively associated with aggression.^9^

#### Combining datasets

##### Combining SNPs, DEGs, CHCs, relatedness, and environmental variables counts in a multinomial regression

In the multinomial logistic regression, we integrated principal component 1 of the CHC analysis, three SNPs, eight gene expression counts, and colony and environmental variables to assess whether they were associated with the three behavioural states (*started aggression*, *reacted aggressively*, *reacted peacefully*). Although CHCs, SNPs, gene expression counts, and environmental variables represent distinct biological and abiotic entities, they have high-dimensional features measured across the same samples and thus share a common statistical role. Moreover, a multinomial regression provides the opportunity to use a unified framework to quantify their joint contribution to categorical outcomes while preserving interpretability.

In total, we tested 24 models (Table S9). Models 1-8 used expression counts of DEGs that were observed in both behavioural comparisons (Figure 3B; Table S3 highlighted cells). Models 9-16 used expression counts of DEGs that were up-regulated in the behavioural comparisons. Models 17-24 used expression counts of DEGs that were down-regulated in the behavioural comparisons. Fitting separate models with increasing number of input variables allowed us to assess if the input variables influence the behavioural states in combination or separately. For example, if some genes are up-regulated in workers that *reacted aggressively* but other genes are up-regulated in workers that *reacted peacefully*, using these genes in combination may lead to false conclusions.

In detail, we tested the following models, namely “intercept-only” models (Models1, 9, 17), models with all three SNP states only (Models 2, 10, 18), models with DEGs that had a log_2_fold value of at least ±0.5 (Models 3, 11, 19), models with all three SNP states and DEGs (log_2_fold of at least ±0.5; Models 4, 12, 20), models with the within-colony relatedness values, standardised air temperature, the first PC from a CHC PCA, site-specific soil nitrogen values, mean annual precipitation, precipitation of the warmest quarter, mean annual temperature, and maximum temperature of the warmest month (“colony and environmental variables”; Models 5, 13, 21), models with all three SNP states and the colony and environmental variables (Models 6, 14, 22), models with DEGs (log_2_fold of at least ±0.5) and colony and environmental variables (Models 7, 15, 23), and, lastly, models with all above-mentioned variables (Models 8, 16, 24).

We compared the model fits using the “anova” function (basic stats package; “Chi-square test”). We further calculated the Akaike Information Criterion for small sample sizes (AICc) of the models (excluding the intercept-only model) using the “aictab” function (“AICcmodavg” package^70^) and the models with the lowest ΔAICc (deltaAICc) values represented the best fitting models. Additionally, we calculated a goodness of fit measure for these models by comparing the fit of observed and expected values. To further test if the used variables are significantly influencing the behavioural states, we manually calculated the *p*-values using a two-tailed Wald Z test (statistical differences represented as ∗∗ = <0.001; ∗∗ = <0.01; ∗ = <0.05). We used the behavioural state *started aggression* as baseline in the logistic regression.

To subsequently test if the behavioural states differ from each other, we compared their means in a pairwise manner. For this, we calculated the marginal means between the behavioural states using the functions “emmeans” and “contrast” (“emmeans” package). These post-hoc tests compared the behavioural states and allowed conducting hypothesis tests to determine whether the differences were statistically significant. We also calculated two pseudo coefficients of determination (R^2^, “Nagelkerke” and “McFadden”) to check how much of the variation is explained by the independent variables. As we used multinomial regressions, the pseudo-R^2^ values were only approximated. We further assessed the significance of the independent variables individually using a likelihood ratio test “lrtest” (“lmtest” package^71^). This test drops the focal variable in the model to assess its impact on the model (i.e., it compares a focal model with the same model by excluding the targeted independent variable). If the model differs significantly, the focal variable is a dominant variable in the model.

## Supplemental information

Document S1. Figures S1–S5, Tables S1–S4 and S6–S20, supplemental materials and methods, and supplemental results

Table S5. Known genes with log2fold change, p-value, corrected p-value and FlyBase name
