# Supplementary material for: Gut-microbiome composition, single nucleotide polymorphisms, and differentially expressed genes correlate with aggression in an ant
Source: iScience. 2026 Aug 24;29(9):117164. doi: 10.1016/j.isci.2026.117164 (PMC13524771; doi:10.1016/j.isci.2026.117164)
Supplement: Supplementary file 1 [file mmc1.pdf]

iScience, Volume *29*

Supplemental information

Gut-microbiome composition, single nucleotide polymorphisms, and differentially expressed genes correlate with aggression in an ant

Patrick Krapf, Francesco Cicconardi, Martin Schilling, Gerhard P. Aigner, Thomas Klammsteiner, Manfred Ayasse, Wolfgang Arthofer, Alexander S. Mikheyev, Florian M. Steiner, and Birgit C. Schlick-Steiner

Supporting Information Text

Supplementary Material and Methods

***Tetramorium alpestre* annotation**

To analyse the RNA samples, we created a new annotation using an existing preliminary annotation of *T. alpestre* and the *T. alpestre* assembly. For this, we merged all transcriptome files into a single file, normalised them using “bbnorm” with a target depth of 50 and a minimum depth of 10, and created a new annotation using “STAR”.[1] Then, we aligned single transcriptomic reads to the new annotation using “STAR”. We calculated counts for differentially expressed genes for each individual using the software “STAR” (command “--quantMode GeneCounts” during the alignment process). For the alignment, we used the reference genome and the new annotation.

Supplementary Results

Recognition assays

To assess whether workers recognise own against alien colony odour,[2] we conducted recognition assays using extracted CHCs from each of the nine colonies ($N_{AntsTested}$=1379). Significantly more workers preferred to reside on their own colony odour (N=502) compared with an alien odour (N=423) or the control with no odour (N=454; multinomial Goodness-of-Fit test assuming the null hypothesis of equal probabilities for each choice; p-value = 0.033).

Differentially expressed genes

In the comparison between ants that *started aggression* and *reacted aggressively*, we found 17,149 DEGs. Of these, 7810 (45.5%) genes were upregulated and 9339 (54.5%) were downregulated in ants that *started aggression* (Fig. S4A, volcano plot - blue dots). Of these 7810 and 9339 genes, 13 and 36 genes were significantly up and downregulated in workers that *started aggression*, respectively (FDR-corrected for multiple testing; Fig. S4, red dots). When comparing ants that *started aggression* with workers that *reacted peacefully*, we found 16,772 genes. Of these, 7436 (44.3%) genes were upregulated, and 9936 (55.7%) were downregulated in workers that *started aggression* (Fig. S4B, volcano plot - blue dots). Of these 7436 and 9936 genes, 28 and 61 genes were significantly up and downregulated, respectively. When comparing ants that *reacted aggressively* versus workers that *reacted peacefully*, we found 17,355 genes with a non-zero count, but zero (0.0%) genes were up or downregulated in workers that *started aggression* (Fig. S4C).

Logistic multinomial regression analyses

Compared with the intercept-only model (Model 1), Models 2-8 significantly explained more variance (Tab. S9). The best model based on the lowest ΔAICc, excluding the intercept-only model, was Model 2 (SNPs only; AICc = 159.92, ΔAICc= 0) followed by Model 4 (SNPs and gene expression; AICc = 164.93, ΔAICc= 5.01; Tab. S10). We calculated a goodness of fit measure for both models by comparing the fit of observed and expected values, and both values displayed a good fit (Model 2: χ^2 = 35.28, df = 2, p-value < 0.001, Residual deviance = 141.95; Model 4: χ^2 = 44.93, df= 4; p-value < 0.001, Residual deviance = 124.56). For Model 2 (SNPs only model), SNP2 and SNP3 significantly influenced the behavioural states (Tab. S11). The odds ratio that SNP2 was associated with the behavioural states was 31.5 times higher for reacting aggressively and 11.8 times higher for reacting peacefully compared to *started aggression* (z-values for *reacted aggressively*: 3.01; *reacted peacefully*: 2.78; Tab. S11). The odds ratio that SNP3 was associated with the behavioural states was 0.1 times lower for reacting aggressively as well as for reacting peacefully compared to *started aggression* (z-values *reacted aggressively*: -3.44; *reacted peacefully*: -2.85; Tab. S11).

For Model 4 (SNPs and DEGs), SNP2, SNP3, and gene *yellow-d2* significantly influenced the behavioural states (Tab. S12). The odds ratio that SNP2 was associated with the behavioural states was 39.2 times higher for reacting aggressively and 11.8 times higher for reacting peacefully compared to *started aggression* (z-values for *reacted aggressively*: 11.51; *reacted peacefully*: 6.09; Tab. S11). The odds ratio that SNP3 was associated with the behavioural states was 0.1 times lower for reacting aggressively and reacting peacefully compared to *started aggression* (z-values *reacted aggressively*: -7.46; *reacted peacefully*: -4.82; Tab. S11). The odds ratio that gene *yellow-d2* was associated with the behavioural states was 1.0 times higher for reacting aggressively and reacting peacefully compared to *started aggression* (z-values *yellow-d2 reacted aggressively*: 2.15; *reacted peacefully*: 2.36; Tab. S11). The calculated pseudo-R^2 values “Nagelkerke” for Model 2 and Model 4 were 0.41 and 0.89, respectively (Tab. S13, for all models).

In the pairwise comparison of the behavioural states (“emmeans” analysis), the means did not differ in either Model 2 or 4 (Tab. S14). In Model 2, comparisons between state *started aggression* and both reactions were borderline significant (estimate *started aggression* vs. *reacted peacefully*: 0.33; df

= 8, t-ratio = 2.59, p-value = 0.07; estimate *started aggression* vs. *reacted peacefully*: 0.35; df = 8, t-ratio = 2.58, p-value = 0.08), but not for *reacted aggressively* vs. *reacted peacefully* (estimate: 0.02; df = 8, t-ratio = 0.21, p-value = 0.98). Further, we used log-likelihood ratio tests to assess the significance of independent variables. For both Models 2 and 4, we tested all variables individually by comparing a full model (e.g., 'SNP1 + SNP2 + SNP3') and a model that lacked the targeted variable (e.g., 'SNP1 + SNP2 + SNP3' with 'SNP1 + SNP2'; SNP3 is the targeted variable). In testing the variables for Models 2 and 4, all models converged. In Model 2, adding SNP2 and SNP3 individually significantly improved the model fit (Tab. S15). For Model 4, adding SNP2 and SNP3 individually as well as adding the gene *yellow-d2* significantly improved the model fit (Tab. S15).

In Models 9-16, we used only upregulated genes. Compared with the intercept-only model (Model 9), Models 10-16 significantly explained more variance (Tab. S9). Model 10 used the same SNPs as Model 2; its results are identical and shown above (Model 2). Model 10 and Model 12 were the best fitting models (ΔAICc = 0 and = 4.63, respectively; model fit Model 12: χ^2 = 43.16, df= 4; p-value < 0.001, Residual deviance = 130.28, AIC = 158.28; Tab. S9). In Model 12, the odds ratio that SNP2 was associated with states *reacted aggressively* and *reacted peacefully* were 32.1 and 11.4 times; and for SNP3 2.2 and 2.1 times fewer, but no gene expression influenced the behavioural states (Tab. S16). The calculated pseudo-R^2 value "Nagelkerke" for Model 12 was 0.50 (Tab. S13). For Model 12, the post-hoc tests ("emmeans" package) revealed that the means of behavioural states *started aggression* and *reacted peacefully* were different (Tab. S16), but no other mean comparison was significant. In the log-likelihood ratio tests, all models converged and revealed that in Model 12, adding SNP2 and SNP3 individually significantly improved the model fit as well as adding the gene *CG34367* (Tab. S15).

In Models 17-24, we only used downregulated genes. Compared with the intercept-only model (Model 17), Models 18-24 significantly explained more variance (Tab. S9). Model 18 used the same SNPs as explanatory variables as Model 2, and the results are shown above. Model 20 and Model 18 were the best fitting models (ΔAICc= 0 and = 1.98, respectively; model fit Model 20: χ^2 = 67.13, df= 4; p-value < 0.001, Residual deviance = 96.79, AIC = 140.79). In Model 20, the odds ratio of being associated with *reacted aggressively* and *reacted peacefully* were 1.6 and 1.6 times higher for SNP1, 54.1 and 31.2 times higher for SNP2, and 0.01 and 0.02 times fewer for SNP3, respectively. Also, several DEGs (i.e., *BicC, CG3238, RFC3*) were associated with the behavioural states (Tab. S17): The odds ratio that gene *BicC* was associated with *reacted aggressively* and *reacted peacefully* was 1.9 and 1.9 times higher, 1.3 and 0.9 times higher and lower, respectively. For gene *CG3238*, it was 1.1 and 1.2 times higher, and 1.3 and 1.3 times higher for gene *RFC3* (12676), respectively. The calculated pseudo-R^2 value "Nagelkerke" for Model 20 was 0.71 (Tab. S13). For Model 20, the calculated post-hoc tests ("emmeans" package) revealed that the means of behavioural states *started aggression* vs. *reacted aggressively* and *started aggression* vs. *reacted peacefully* were different (Tab. S14), but the mean between *reacted aggression* vs. *reacted peacefully* was not. In the log-likelihood ratio tests, all models converged and revealed that in Model 20, adding SNP2 and SNP3 individually as well as the genes *BicC, Pif1*, and *RFC3* significantly improved the fit of the model (Tab. S15).

Gene-enrichment analyses with known genes

Using g:Profiler, we pairwise compared all behavioural states. In the comparison *started aggression* vs. *reacted aggressively*, eight and nine up and downregulated genes were used, respectively. For upregulated genes in *started aggression* vs. *reacted aggressively*, 26 biological processes and five molecular functions were enriched, which can be summarised into enzyme activities, cellular processes, developmental processes, metabolic processes, cellular structural organisation, and biological regulation. For downregulated genes, ten biological processes, six molecular functions, and five cellular components that were enriched. These can be broadly summarised into enzyme activities, transporter activities, receptor activities, protein binding, cellular processes, signalling pathways, regulation of cellular processes, cellular localisation and responses, and axon guidance. In the comparison *started aggression* vs. *reacted peacefully*, we used 25 and 20 up and downregulated genes, respectively. For upregulated genes, we found six cellular components that were enriched, which can be summarised in cellular structures, components, and extensions. For downregulated genes, we found 13 molecular functions and three cellular components. These can be summarised in ion-channel activities, enzyme activities, RNA and protein binding, and cellular components. In the comparison *reacted aggressively* vs. *reacted peacefully*, no genes were differently expressed, and no gene-enrichment analysis could be conducted.

To check whether the same or different genes were up and downregulated genes in the three behavioural states, we created Venn diagrams (<0.05 FDR-corrected genes with known functions were used). In total, 30 upregulated genes were used: five of those genes (16.6%; *CG3800, CG3902, CDase, Rhp,* and *Moe*; Tab. S5 highlighted in dark yellow) were only found in the comparison *started aggression* vs. *reacted aggressively*, 22 genes (73.3%; *CG34367, CG13625, CG3655, apolpp, CG14687, CG3655, Gat, Sur-8, mRpL9, CG9175, CG6656, Phm, Socs16D, Vav, CG3860, CG32225, CG9426, alph,*

CG16974, CG10483, AP-2alpha, and *bchs*; Tab. S5 highlighted in light yellow) only in the comparison *started aggression* vs. *reacted peacefully*, and three (10.0%; genes *CG3061, svr,* and *Syt4*; Tab. S5 highlighted in yellow and bold) in both comparisons *started aggression* vs. *reacted aggressively* and *started aggression* vs. *reacted peacefully*. We did not find any genes in the comparison *reacted aggressively* vs. *reacted peacefully*. The log_2fold change values of these genes ranged between 3.19 and 0.12 for *started aggression* vs. *reacted peacefully* and between 0.50 and 0.11 for *started aggression* vs. *reacted peacefully* (Tab. S5).

In total, 28 downregulated genes were used in the Venn diagrams: eight of them (28.6%; *BicC, Pif1 (CG3238), Exo84, PlexA, Tret1-1, Taf5, Doa,* and, *Vps35*; Tab. S5 highlighted in dark blue; Fig. 3B) were in the comparison *started aggression* vs. *reacted aggressively*, 19 in the comparison *started aggression* vs. *reacted peacefully* (67.9%; *CG3822, Rdl, RFC3, CG10431, Cdep, Dscam1, CG7492, CG6910, snRNP-U1-70K, CG31550, l(1)G0196, CG9346, CG32486, agt, Gcn5, baz, CG13366, U2af50,* and *CG8108*; Tab. S5 highlighted in light blue); and one (3.5%; *yellow-d2*; Tab. S5 highlighted in blue and bold) was found in both comparisons *started aggression* vs. *reacted aggressively* and *started aggression* vs. *reacted peacefully*. The log_2fold changes of these genes ranged between -3.06 and -0.15 for *started aggression* vs. *reacted peacefully* and between -1.60 and -0.15 for *started aggression* vs. *reacted peacefully* (Tab. S5). Of these genes, two were highly expressed: gene *CG3800* was highly upregulated (log_2fold change = 2.48) and gene *BicC* was highly downregulated (log_2fold change = -3.06). The above-mentioned genes were used for the multinomial regression analyses.

Supplementary Discussion

Six downregulated genes linked to synaptic functions

BicC encodes an RNA-binding protein that is influencing protein translation during embryonic development (FlyBase). An upregulation of *BicC* is associated with chronic stress and depression in mice and other animals.[3-5] In contrast, a downregulation of *BicC* activates the Wnt pathway,[6] which is a conserved pathway regulating, among others, cell fate determination and organogenesis during development[7]. For example, a downregulation of *BicC* restores normal pleasure-seeking behaviour in depressive rats[4] and may affect synaptic plasticity.[5] We thus speculate that its downregulation is associated with more active workers. Overall, downregulating *BicC* might lead more aggression in ants.

Exo84 belongs to the Exocyst complex and is vital for secretion in organisms ranging from yeasts to mammals.[8] It is enriched in the brain[9] and has been predicted to be important in cell signal transduction pathways and the cytoskeleton network.[8] *Exo84* is vital for multiple developmental processes, such as epithelial polarity and apical protein localisation in *Drosophila*[10] and neurite differentiation.[11] Its downregulation may cause a reduction in synaptic transmission.

The gene *KaiR1d* is a glutamate receptor and, among others, essential for the calcium sensitivity of baseline synaptic transmission.[12] Its downregulation possibly diminishes the calcium sensitivity and may result in altered neurotransmitter release dynamics and possibly influence the behavioural states of the workers tested.

The gene *Rdl* (*Resistant to dieldrin*) encodes an abundant chloride channel, which is a receptor for gamma-amino butyric acid (GABA) thus mediating GABA inhibitory neurotransmission.[13] It is, for example, expressed in specific brain structure of *Drosophila* (mushroom bodies) and essential for courtship behaviour in *Drosophila*[14] as well as olfactory learning in insects.[13] Notably, flies with a *Rdl* knock-down have an increased memory acquisition[13] and also display higher aggression levels in courtship behaviour.[14] This downregulation may thus be one factor explaining why workers *started aggression*.

The gene *yellow-d2* belongs to the highly conserved *yellow*-gene family, which has various functions linked to pigmentiation.[15] *Yellow* genes, in general, are known to play an essential role in male courtship behaviour in *Drosophila*[16] and are assumed to influence nearby cells such as dopamine receptors[17] through dopamine receptor downstream signalling. Notably, *Drosophila* males with mutant yellow genes display a reduced courtship behaviour.[16] This may indicate that downregulation of *yellow-d2* could possibly impact aggressive behaviour.

The gene *PlexA* is a conserved functional receptor for semaphorins, for example, in *Drosophila,*[18] and crickets.[19] Semaphorins are membrane proteins of the nervous system[18] binding to plexins. Most effects of downregulated *PlexA* in adult organisms are unknown but may result in an increase of excitatory synapses and in the frequency of synaptic signals[20] potentially contributing to quicker reaction.

Further, we found two downregulated genes associated with DNA repair and replication. One gene is *Pif1*, which is part of DNA helicases, which are evolutionarily conserved among prokaryotes and eukaryotes.[21] DNA helicases are important for DNA recombination, repair, replication, transcription, and translation.[22] The other gene is *RFC3*, which encodes the Replication Factor C Subunit 3, which is part of the *RFC* (Replication Factor C complex). It is essential for DNA replication[23] and associated with human cancer development.[24]

Figures

Figures start on the next page

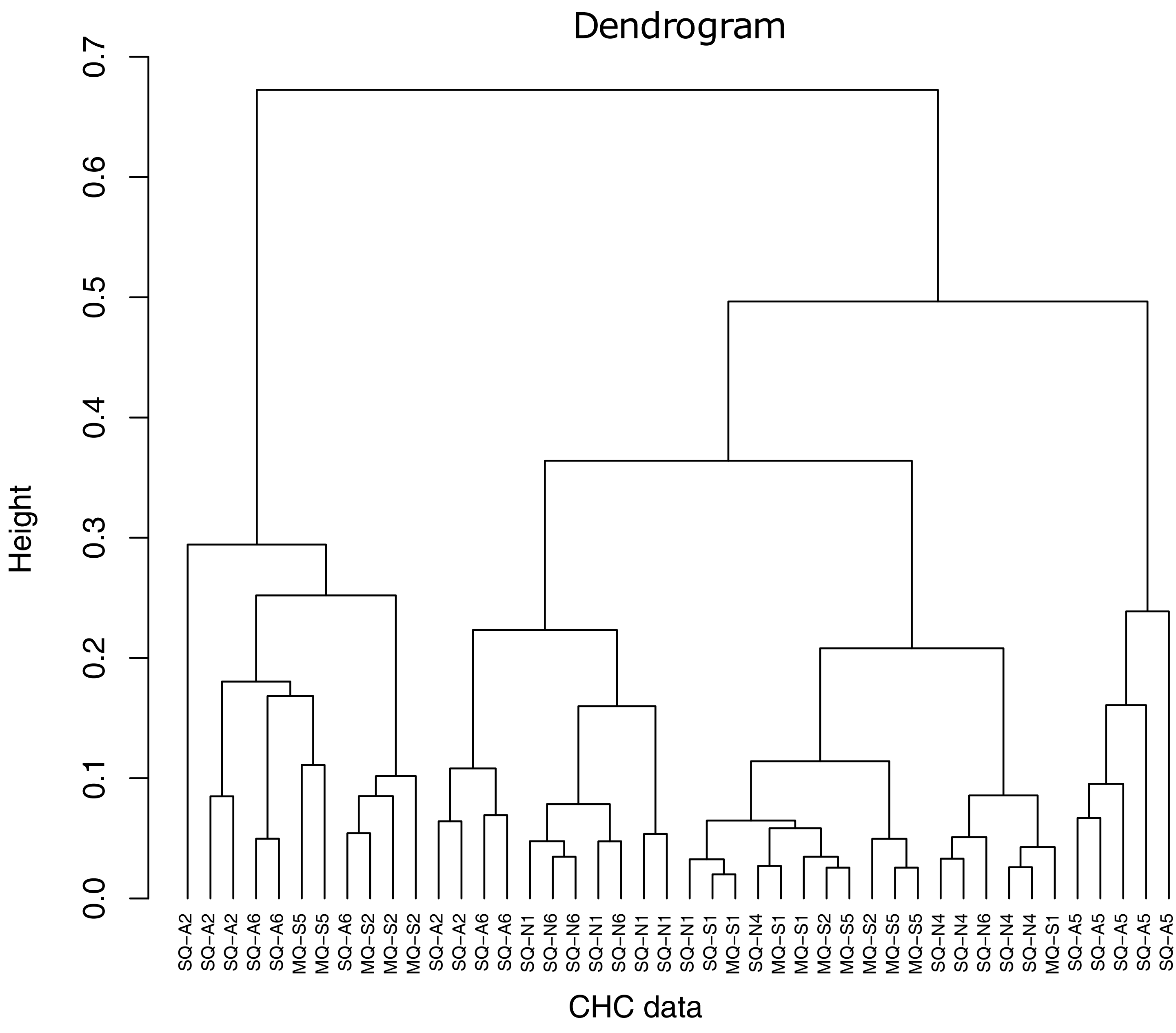

Dendrogram
Height
0.0
0.1
0.2
0.3
0.4
0.5
0.6
0.7
SQ−A2
SQ−A2
SQ−A2
SQ−A6
SQ−A6
MQ−S5
MQ−S5
SQ−A6
MQ−S2
MQ−S2
MQ−S2
SQ−A2
SQ−A2
SQ−A6
SQ−A6
SQ−N1
SQ−N6
SQ−N6
SQ−N1
SQ−N6
SQ−N1
SQ−N1
SQ−N1
SQ−S1
MQ−S1
SQ−N4
MQ−S1
MQ−S1
MQ−S2
MQ−S5
MQ−S2
MQ−S5
MQ−S5
SQ−N4
SQ−N4
SQ−N6
SQ−N4
SQ−N4
MQ−S1
SQ−A5
SQ−A5
SQ−A5
SQ−A5
SQ−A5
CHC data

Fig. S1. Hierarchical cluster analysis using CHC data from each colony. Samples from SQ-N and SQ-S had similar CHC profiles and partially clustered together ($N_{workers}$=44). Samples from SQ-A5 were more similar to SQ-N and MQ-N colonies than to SQ-A2 and SQ-A6. The latter two clustered closely to MQ-N2 and MQ-N5 as well as SQ-N1 and SQ-N6. Note: SQ-A = single-queened and aggressive, SQ-N = single-queened and non-aggressive, MQ-N = multiple-queened and non-aggressive.

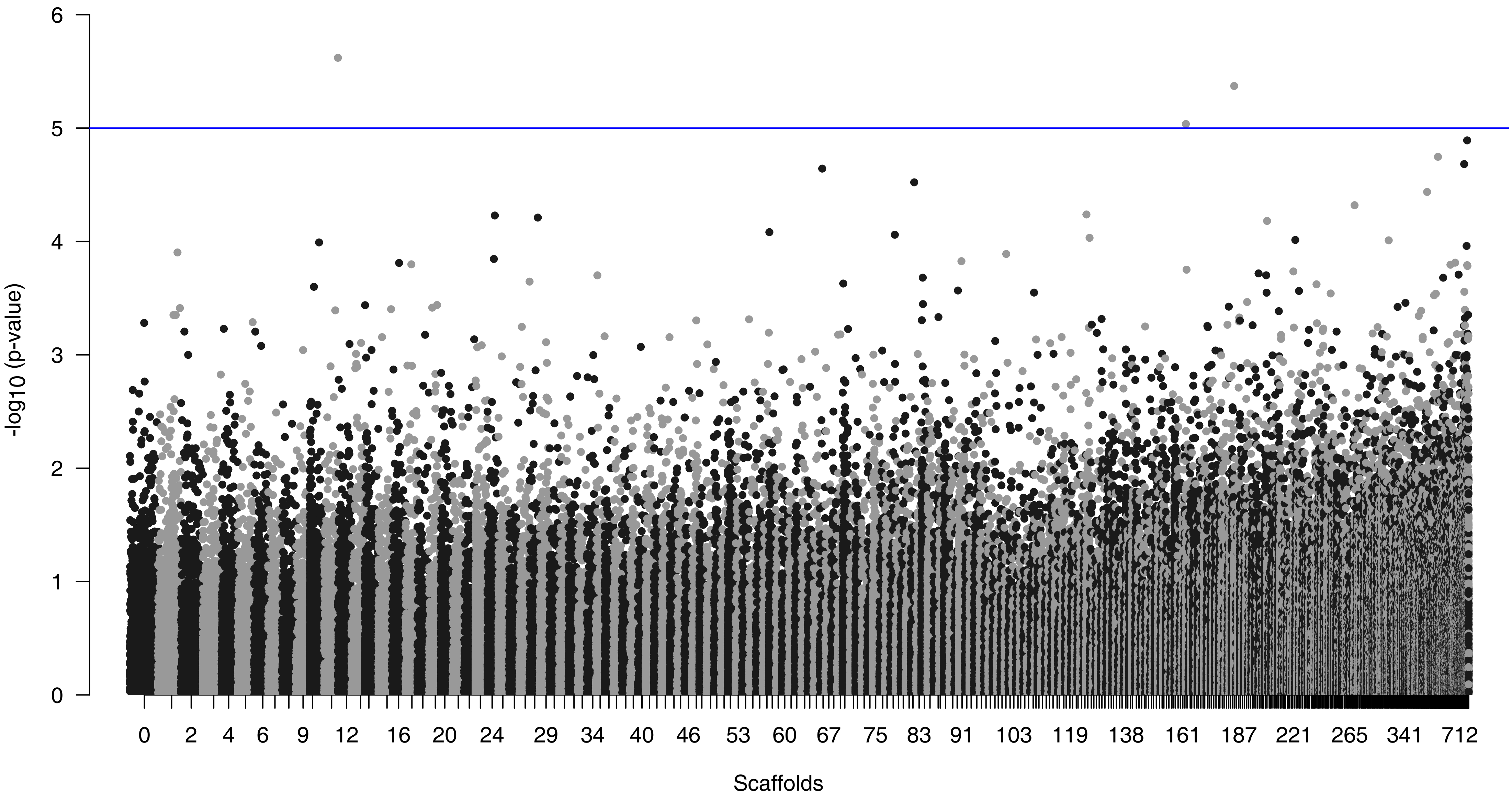

-log10 (p-value)
6
5
4
3
2
1
0
0 2 4 6 9 12 16 20 24 29 34 40 46 53 60 67 75 83 91 103 119 138 161 187 221 265 341 712
Scaffolds

Fig. S2. Manhattan plot of all scaffolds. The blue line represents the suggestive line, and three SNPs are above the suggestive line, namely SNP1 at scaffold 11 - site 2457277, SNP2 at scaffold 165 - site 28302, and SNP3 at scaffold 185 - site 110741, site in gene *gastrulation-defective*.

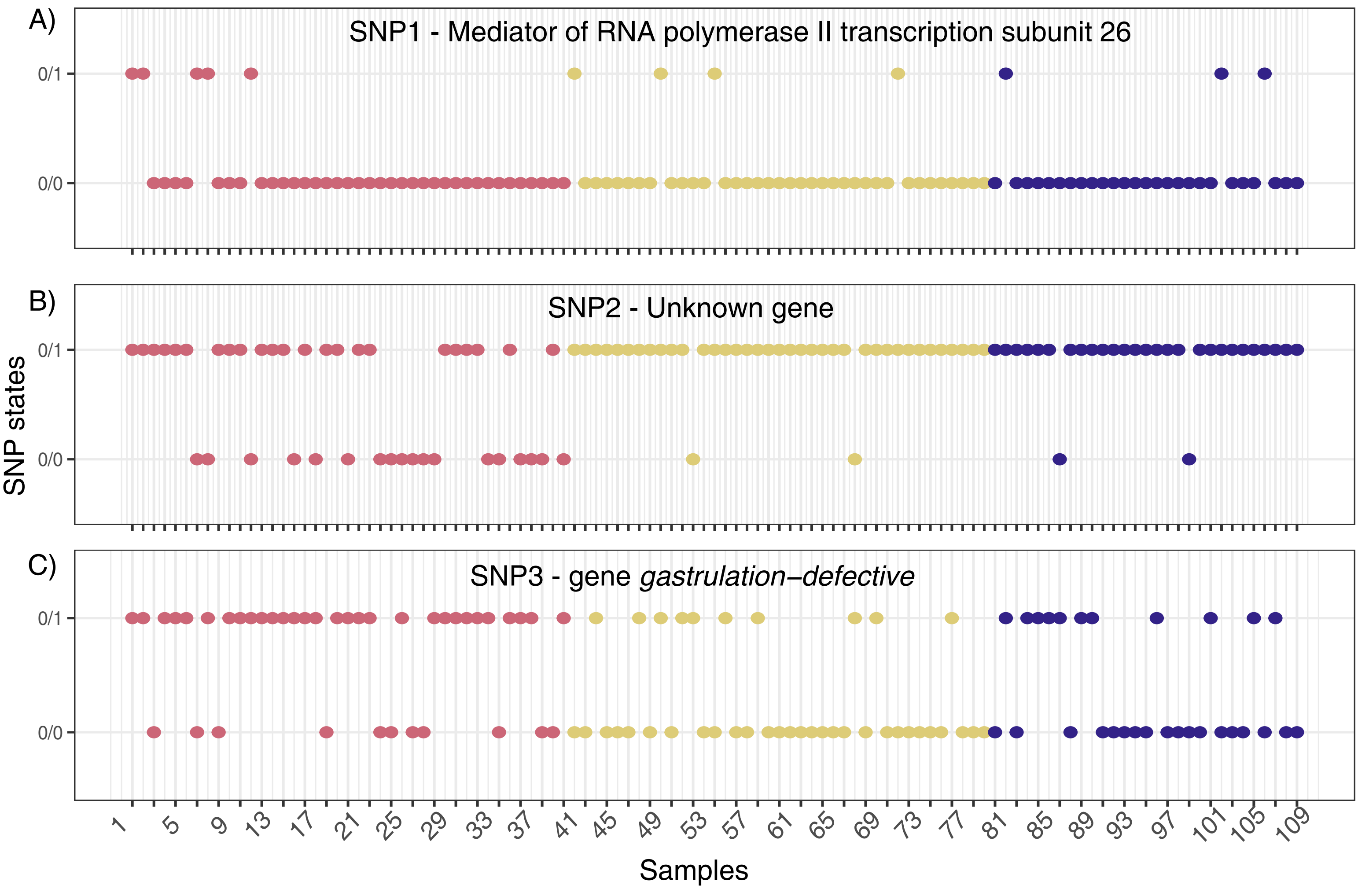
A)
SNP1 - Mediator of RNA polymerase II transcription subunit 26
B)
SNP2 - Unknown gene
C)
SNP3 - gene *gastrulation–defective*
0/1
0/0
SNP states
Samples
1
5
9
13
17
21
25
29
33
37
41
45
49
53
57
61
65
69
73
77
81
85
89
93
97
101
105
109

Fig. S3. Allelic SNPs states of each worker represented for each of the three SNPs. The samples from 1 to 109 representing all workers, the numeric combinations "0/0" represents homozygous alleles or "0/1" heterozygous alleles). A) The first panel represent the gene "Mediator of RNA polymerase II transcription subunit 26". Here, 36 out of 41 (88%) workers that *started aggression*, 35 out of 39 workers (90%) that *reacted aggressively*, and 26 out of 29 (90%) workers that *reacted peacefully* were homozygous for the reference allele. This indicates that this gene is not a strong candidate for the behavioural states. B) The second panel represents an unknown gene. Here, 18 out of 41 (43%) workers that *started aggression* were homozygous in the SNP state, while 2 out of 39 (5%) workers that *reacted aggressively* and 2 out of 29 (7%) workers that *reacted peacefully* were homozygous in the SNP state, respectively. Although this is a currently unknown, this gene could be a driver for the behavioural states observed. C) The third panel represents the gene *gastrulation-defective* (*gd*). Here, 30 out of 41 (73%) workers that *started aggression* were heterozygous for the SNP state, while 10 out of 39 (26%) workers that *reacted aggressively* and 11 out of 29 (38%) workers that *reacted peacefully* were heterozygous for the behavioural state. This suggests that heterozygosity in the gene *gd* could positively influence the start of aggressive behaviour in workers.

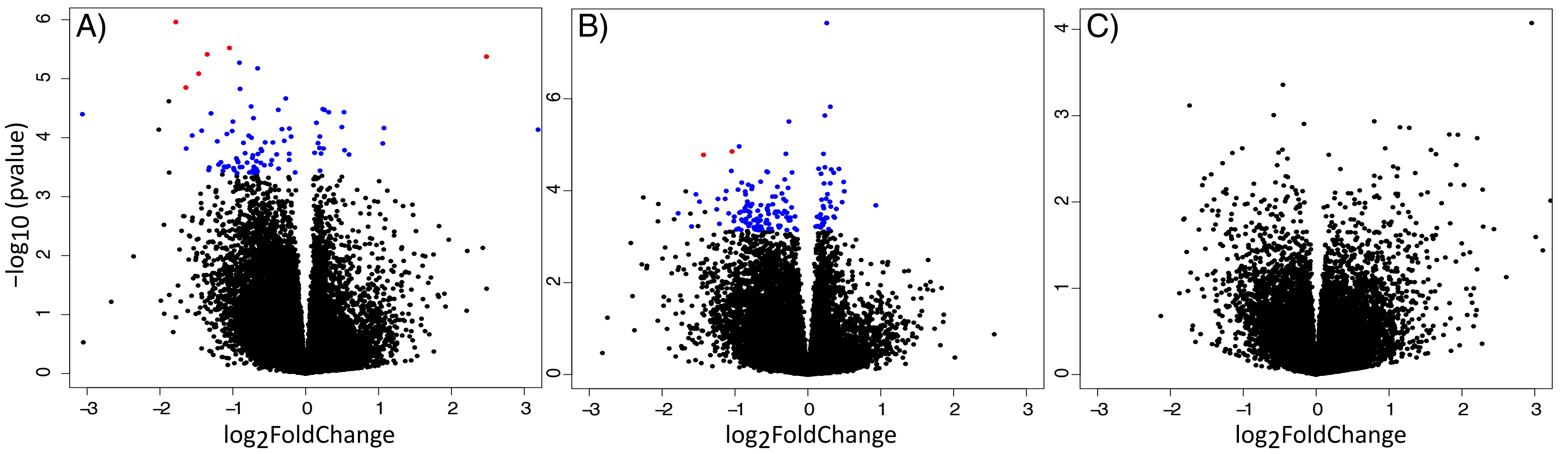

A)
B)
C)
−log10 (pvalue)
log2FoldChange
log2FoldChange
log2FoldChange

Fig. S4. Gene expression volcano plots from 82 workers for the three behavioural states. Differentially expressed genes A) between behavioural states *started aggression* vs. *reacted aggressively* are displayed, B) between *started aggression* vs. *reacted peacefully*, and C) between *reacted aggressively* vs. *reacted peacefully*. Red dots represent statistically significant (corrected for multiple testing using FDR with <0.05) genes with a $\log_2$fold change smaller than -1 or larger than 1, while blue dots represent statistically significant genes (not corrected for multiple testing). In total, 136 (1%) upregulated and 437 (2.5) downregulated genes were discovered in the comparison *started aggression* vs. *reacted aggressively* (total, 17153 genes) and 146 upregulated and 324 downregulated genes in *started aggression* vs. *reacted peacefully* (total, 16776 genes). However, no genes were differentially expressed in the comparison *reacted aggressively* vs. *reacted peacefully*. Of these DEGs, 761 out of 1043 (73%) genes were downregulated in *started aggression* vs. *reacted aggressively* and *started aggression* vs. *reacted peacefully*, while 282 out of 1043 (27%) genes were upregulated.

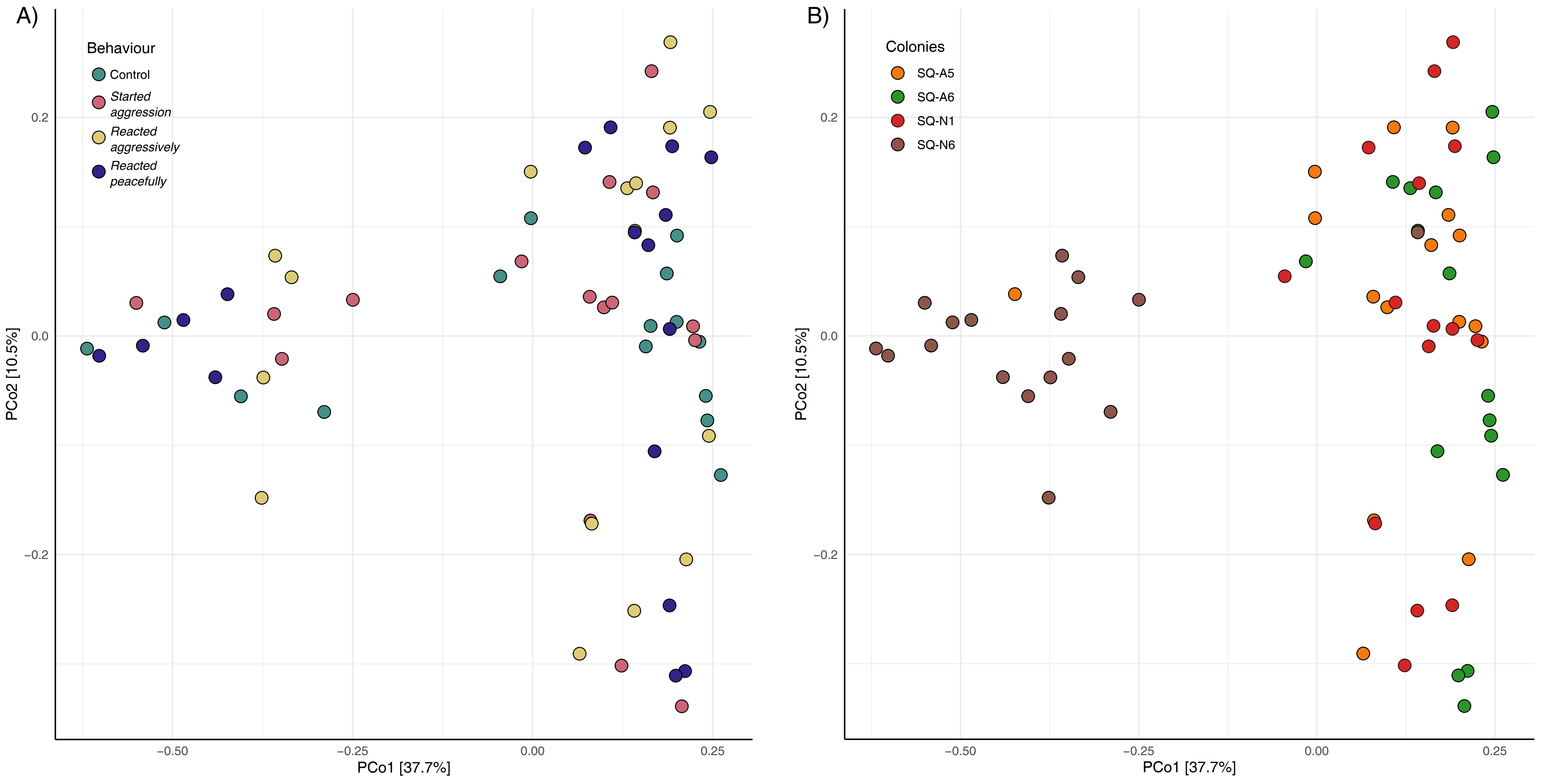
A)
Behaviour
Control
Started aggression
Reacted aggressively
Reacted peacefully
PCo2 [10.5%]
PCo1 [37.7%]
0.2
0.0
−0.2
−0.50
−0.25
0.00
0.25
B)
Colonies
SQ-A5
SQ-A6
SQ-N1
SQ-N6
PCo2 [10.5%]
PCo1 [37.7%]

Fig. S5. Principal Coordinate Analysis (PCoA) of the microbiome data. In A), all samples including the control samples are displayed ($N_{samples}$=65). There was no apparent clustering across the directly frozen control samples (i.e., workers frozen in the field) and the other samples (i.e., workers frozen after laboratory maintenance and aggression assays). In B), the same samples are displayed as in A) but coloured based on their colony origin. Here, the samples cluster based on their microbiome, with SQ-N6 clustering on the left side of the x-axis and all other samples clustering together. Note: SQ-A = single-queened and aggressive, SQ-N = single-queened and non-aggressive.

Table S1. The countries, locations, colony IDs, GPS coordinates, selected behavioural states, and the number of workers used for genomic and transcriptomic analyses.

**Country, location**	**Population abbreviation**	**Colony ID**	**GPS coordinates (Latitude, Longitude)**	***Started aggression***	***Reacted aggressively***	***Reacted peacefully***	**Number of workers**	**Total number of workers used for WGS and expression analyses**
Italy, Penser Joch	SQ-A	SQ-A2	46.81403, 11.44196	2	1	2	5	31
		SQ-A5	46.81440, 11.44244	6 [4]	3 [4]	1 [4]	10 [12]	
		SQ-A6	46.81483, 11.44271	2 [4]	9 [4]	5 [4]	16 [12]	
Austria, Kuehtai	SQ-N	SQ-N1	47.21915, 11.07514	1 [4]	5 [4]	4 [4]	10 [12]	28
		SQ-N4	47.21926, 11.07610	6	3	1	10	
		SQ-N6	47.21920, 11.07661	3 [4]	3 [4]	2 [5]	8 [13]	
Austria, Mussen	MQ-N (additional workers)	MQ-S1	46.7071, 12.92747	5 (5)	2 (3)	3 (1)	10 (9)	26 (24)
		MQ-S2	46.70754, 12.92800	2 (5)	2 (2)	4 (1)	8 (8)	
		MQ-S5	46.7098, 12.92262	2 (2)	4 (1)	2 (4)	8 (7)	
	Total number of workers (incl. additional workers from MQ-N)			31 (12)	29 (6)	25 (6)		85 (109)

Note: (Numbers in brackets) represent additional samples used for WGS; [Numbers in squared brackets] represent samples used for microbiome analyses. SQ-A = single-queened and aggressive, SQ-N = single-queened and non-aggressive, MQ-N = multiple-queened and non-aggressive

Table S2. CHC compounds for each colony

Population →	SQ-A			SQ-N			MQ-N		
**Colony →**	**SQ-A2**	**SQ-A5**	**SQ-A6**	**SQ-N1**	**SQ-N4**	**SQ-N6**	**MQ-N1**	**MQ-N2**	**MQ-N5**
**CHC compound ↓**									
Dodecane	0.00940	0.00777	0.00871	0.00776	0.00744	0.00839	0.00746	0.00832	0.00751
Tridecene*	0.00251	0.00102	0.00189	0.00062	0.00041	0.00065	0.00072	0.00148	0.00167
Tridecane	0.01188	0.00925	0.00871	0.00880	0.00965	0.00976	0.01484	0.01040	0.00945
Tetradecene*	0.00436	0.00143	0.00303	0.00104	0.00113	0.00164	0.00112	0.00201	0.00268
Tetradecane	0.01103	0.00830	0.00879	0.00665	0.00661	0.00728	0.00688	0.00723	0.00755
Pentadecene1*	0.02174	0.00865	0.00975	0.00214	0.00418	0.00898	0.00249	0.00439	0.00511
Pentadecene2*	0.00746	0.00548	0.00437	0.00120	0.00157	0.00300	0.00105	0.00130	0.00177
Pentadecene3*	0.00244	0.00169	0.00200	0.00150	0.00217	0.00305	0.00157	0.00110	0.00173
Pentadecene4*	0.00785	0.00199	0.00403	0.00112	0.00101	0.00227	0.00136	0.00298	0.00351
Pentadecane	0.07663	0.05245	0.01739	0.00766	0.02050	0.02342	0.00834	0.01254	0.01121
Hexadecane	0.01308	0.00438	0.00910	0.00390	0.00352	0.00540	0.00447	0.00568	0.00716
Tetradecanal	0.00397	0.00107	0.00304	0.00055	0.00071	0.00087	0.00040	0.00260	0.00236
Heptadecene1*	0.03839	0.01464	0.01919	0.00384	0.01061	0.01170	0.00446	0.00846	0.00662
Heptadecene2*	0.00451	0.00271	0.00579	0.00206	0.00516	0.00508	0.00255	0.00107	0.00221
Heptadecene3*	0.00695	0.00153	0.00582	0.00065	0.00059	0.00148	0.00119	0.00313	0.00382
Heptadecene4*	0.00331	0.00347	0.00481	0.00557	0.00258	0.00565	0.00242	0.00303	0.00231
Heptadecane	0.01347	0.00727	0.01444	0.00464	0.00660	0.00744	0.00559	0.00539	0.00818
Unknown	0.00380	0.00120	0.00432	0.00150	0.00147	0.00204	0.00212	0.00253	0.00284
Octadecane	0.02126	0.00402	0.01849	0.00391	0.00312	0.00579	0.00520	0.00775	0.01062
Hexadecanal	0.02890	0.00243	0.00980	0.00075	0.00087	0.00272	0.00106	0.00930	0.00916
Unknown	0.00223	0.00158	0.00208	0.00181	0.00145	0.00145	0.00181	0.00166	0.00191
Unknown	0.00817	0.00106	0.00468	0.00068	0.00150	0.00077	0.00111	0.00117	0.00386
Heptadecanal	0.00506	0.00386	0.00418	0.00445	0.00406	0.00391	0.00362	0.00418	0.00436
Eicosane	0.01937	0.00203	0.01341	0.00203	0.00158	0.00274	0.00238	0.00632	0.00860
Octadecanal	0.02268	0.00193	0.01491	0.00068	0.00046	0.00177	0.00095	0.00901	0.01026
Heneicosane	0.00585	0.00453	0.00407	0.00324	0.00250	0.00300	0.00368	0.00318	0.00417
Docosane	0.00740	0.00847	0.00552	0.00473	0.00265	0.00345	0.00453	0.00372	0.00495
Z-9-Tricosene	0.04163	0.00980	0.02413	0.00648	0.00271	0.00712	0.00425	0.01436	0.02199
Z-7-Tricosene	0.01966	0.01232	0.01301	0.00773	0.00297	0.00669	0.00516	0.00876	0.01480
Tricosane	0.12803	0.21006	0.11244	0.09078	0.03925	0.07168	0.06459	0.06015	0.08622
11-Methyltricosane	0.01105	0.00514	0.00629	0.02042	0.00819	0.01382	0.00312	0.00383	0.00477
3-Methyltricosane	0.12983	0.06860	0.10582	0.06173	0.02811	0.04804	0.03417	0.06900	0.05430
Tetracosane	0.01867	0.02390	0.01766	0.01738	0.00981	0.01445	0.01217	0.01375	0.01550
Pentacosene*	0.00208	0.00214	0.00366	0.00073	0.00082	0.00084	0.00254	0.00259	0.00377
Pentacosene*	0.00624	0.00311	0.00725	0.00289	0.00158	0.00303	0.00210	0.00439	0.00345
Z-11-Pentacosene	0.00413	0.00099	0.00372	0.00166	0.00040	0.00131	0.00071	0.00092	0.00252
Z-9-Pentacosene	0.13014	0.07775	0.13584	0.03712	0.01744	0.03760	0.02567	0.09895	0.12322
Pentacosene*	0.04894	0.03929	0.05422	0.03390	0.01505	0.03339	0.01945	0.04571	0.04144
Pentacosane	0.15546	0.24542	0.17864	0.21834	0.12158	0.17864	0.13924	0.14155	0.17347
13-Metylpentacosane	0.03780	0.01397	0.01946	0.08155	0.04394	0.07681	0.02426	0.03575	0.03306
3-Methylpentacosane	0.01838	0.01289	0.01860	0.00843	0.00740	0.01153	0.00901	0.02069	0.01202

Hexacosane	0.01029	0.01507	0.01300	0.01191	0.01073	0.01233	0.00897	0.01157	0.01019
Z-11Heptacosene	0.00369	0.00145	0.00361	0.00197	0.00141	0.00197	0.00029	0.00076	0.00268
-Z9-Heptacosene	0.01286	0.00720	0.01370	0.00441	0.00271	0.00553	0.00362	0.01475	0.00943
Z-7-Heptacosene	0.01123	0.00570	0.01140	0.00263	0.00185	0.00338	0.00380	0.00994	0.00483
Heptacosene*	0.00285	0.00313	0.00231	0.00128	0.00027	0.00130	0.00063	0.00120	0.00205
Heptacosane	0.04008	0.06140	0.04377	0.04625	0.03695	0.05459	0.03757	0.03869	0.03708
13-Methylheptacosane	0.00886	0.00562	0.00618	0.01682	0.01304	0.01899	0.00522	0.00949	0.00617
Unknown	0.00792	0.01089	0.00819	0.00461	0.00603	0.00861	0.00956	0.01337	0.00935
Octacosane	0.00670	0.00894	0.01024	0.00672	0.00758	0.00623	0.00500	0.00728	0.00612
Unknown	0.00327	0.00281	0.00254	0.00202	0.00213	0.00264	0.00237	0.00425	0.00276
Nonacosen*	0.00782	0.00546	0.00563	0.00602	0.00468	0.00766	0.00608	0.01110	0.00492
Nonacosene*	0.00639	0.00510	0.00839	0.00829	0.00688	0.00909	0.00996	0.01055	0.00608
Nonacosene*	0.00442	0.00401	0.00406	0.00492	0.00419	0.00674	0.00383	0.00822	0.00467
Z-11-Nonacosene	0.00416	0.00230	0.00362	0.00382	0.00412	0.00397	0.00294	0.00585	0.00286
Z-7-Nonacosene	0.00127	0.00126	0.00702	0.00126	0.00103	0.00156	0.00145	0.00384	0.00144
Nonacosene*	0.01139	0.00943	0.01172	0.01144	0.00961	0.01498	0.00954	0.02172	0.01003
Nonacosane	0.01334	0.01647	0.00464	0.01162	0.01117	0.01494	0.00893	0.00955	0.00831
Triacontane	0.00567	0.00664	0.00689	0.00591	0.00673	0.00549	0.00400	0.00574	0.00570
Hentriaconane	0.00700	0.00714	0.00744	0.00453	0.00524	0.00616	0.00267	0.00452	0.00448
Dotriaconane	0.00363	0.00405	0.00356	0.00213	0.00309	0.00262	0.00147	0.00346	0.00256
Tritriacontane	0.00804	0.00616	0.00826	0.00231	0.00288	0.00498	0.00294	0.00399	0.00507
Pentatriacontane	0.00656	0.00203	0.00933	0.00042	0.00099	0.00279	0.00130	0.00212	0.00339

Arrows indicate the direction to read the table. The asterisk (*) represents unknown double bond position; SQ-A = single-queened and aggressive, SQ-N = single-queened and non-aggressive, MQ-N = multiple-queened and non-aggressive colonies.

Table S3. Mean within-colony relatedness and number of queens calculated based on microsatellite genotyping

Population (N)	Colony	Queller and Goodnight[25] relatedness based on microsatellite data	Number of queens (Pamilo 1991)[26] based on microsatellite data	Manichaikul et al. 2010[27] relatedness based on whole-genome data	Number of queens (Pamilo 1991)[26] based on whole-genome data
SQ-A (N = 31)	SQ-A2	0.725	1.05	0.58	1.38
	SQ-A5	0.693	1.11	0.58	1.39
	SQ-A6	0.727	1.04	0.57	1.43
SQ-N (N= 28)	SQ-N1	0.768	0.97	0.63	1.25
	SQ-N4	0.797	0.92	0.63	1.25
	SQ-N6	0.771	0.96	0.62	1.27
MQ-N (N = 50)	MQ-N1	0.456	1.86	0.32	2.79
	MQ-N2	0.385	2.26	0.33	2.73
	MQ-N5	0.361	2.44	0.33	2.66

N = number of samples. Relatedness was calculated using 12 workers per colony and whole-genome sequencing data using 109 individuals, in total). The equation to calculate the number of queens is: (3-r)/(3r). SQ-A = single-queened and aggressive, SQ-N = single-queened and non-aggressive, MQ-N = multiple-queened and non-aggressive

Table S4. Count data of the SNP alleles that were homozygous and heterozygous for the reference genome

**SNP**	**Reference or alternative allele**	***Started aggression***	***Reacted aggressively***	***Reacted peacefully***	**p-value pairwise comparisons "*Started aggression* vs. *reacted aggressively*"**	**p-value pairwise comparisons "*Started aggression* vs. *reacted peacefully*"**	**p-value pairwise comparisons "*Reacted aggressively* vs. *reacted peacefully*"**	**Cramér's V effect size**
SNP 1	Reference	36	35	26	1.00	1.00	1.00	0.03
	Alternative	5	4	3				
SNP 2	Reference	18	2	2	<0.001	0.002	1.00	0.46
	Alternative	23	37	27				
SNP 3	Reference	11	29	18	<0.001	0.013	0.304	0.42
	Alternative	30	10	11				

Similar as depicted above but represented are the exact counts of individuals with reference or alternative alleles. SNP2 is more frequently present in individuals that reacted rather than *started aggression* and SNP3 is more frequently present in individuals that either *started aggression* or *reacted aggressively*. Note: SNP1 = predicted mediator of RNA polymerase; SNP2 = unknown gene; SNP3 = *gastrulation-defective* (*gd*).

Table S6. Rarefied OTU counts for the control and each behavioural state, and their frequency

**Genus or order**	**Sum control**	**Sum *started aggression***	**Sum *reacted aggressively***	**Sum *reacted peacefully***	**Sum without control**	**Frequency [%]**
Acetobacter	627	886	844	894	2624	0.7%
Bacteroides	21002	25814	24398	23800	74012	20.7%
Enterococcus	1853	4264	29832	6454	40550	11.3%
Fusobacterium	937	654	331	678	1663	0.5%
Lactobacillus	14481	22715	20986	22923	66624	18.6%
Megamonas	2826	6570	6841	5806	19217	5.4%
Prevotella	7791	18659	23139	19154	60952	17.0%
Pseudomonas	45271	23942	35009	31914	90865	25.4%
Rhizobiales	282	342	348	325	1015	0.3%
**Total**	95070	103846	141728	111948	357522	100%

OTUs of nine different genera or order are presented above which have been associated with behaviour in various studies (see main text). The frequency of each genus or order is presented as a percentage of the total counts of these OTUs

Table S7. Counts of the behavioural state presented for the nine selected OTUs

**Genus**	**Species OTU**	**Counts total**	**Count control**	**Count *Started aggression***	**Count *Reacted aggressively***	**Count *Reacted peacefully***
Bacteroides	*B. salanitronis* OTU 1598	133	24	67	30	12
	Bacteroides sp. OTU 22324	1018	335	131	278	274
Lactobacillus	*L. mucosae* OTU 813	233	56	73	70	34
	Lactobacillus sp. OTU 21141	374	84	87	78	125
Prevotella	*Prevotella* sp. OTU 377	407	4	333	19	51
	Prevotella bacterium OTU 1887	1055	55	754	106	140
	Prevotella sp. OTU 20448	1118	408	395	127	188
Pseudomonas	*P. psychrotolerans* OTU 366	314	33	134	102	45
	Pseudomonas sp. OTU 2442	192	33	95	30	34

Based on the multinomial logistic regressions, nine OTUs were associated with the behavioural states. Their counts are presented above.

Table S8. Behavioural states comparisons for each OTU count

Genus	Species OTU	Comparison	Log estimate	SE	Z-Ratio	p-value
Bacteroides	*Bacteroides* sp. OTU 1598	***started aggression* vs. *reacted aggressively***	**0.80**	**0.22**	**3.66**	**<0.001**
		started aggression* vs. *reacted peacefully	**1.72**	**0.31**	**5.49**	**<0.001**
		reacted aggressively* vs. *reacted peacefully	**0.92**	**0.34**	**2.68**	**0.020**
	Bacteroides sp. OTU 22324	***started aggression* vs. *reacted aggressively***	**-0.75**	**0.11**	**-7.10**	**<0.001**
		started aggression* vs. *reacted peacefully	**-0.74**	**0.11**	**-6.95**	**<0.001**
		reacted aggressively vs. *reacted peacefully*	0.01	0.09	0.17	0.984
Lactobacillus	*Lactobacillus mucosae* OTU 813	*started aggression* vs. *reacted aggressively*	0.04	0.17	0.25	0.966
		started aggression* vs. *reacted peacefully	**0.76**	**0.21**	**3.68**	**<0.001**
		reacted aggressively* vs. *reacted peacefully	**0.72**	**0.21**	**3.45**	**0.002**
	Lactobacillus sp. OTU 21141	*started aggression* vs. *reacted aggressively*	0.11	0.16	0.70	0.763
		started aggression* vs. *reacted peacefully	**-0.36**	**0.14**	**-2.60**	**0.026**
		reacted aggressively* vs. *reacted peacefully	**-0.47**	**0.14**	**-3.27**	**0.003**
Prevotella	*Prevotella* sp. OTU 377	***started aggression* vs. *reacted aggressively***	**2.86**	**0.24**	**12.14**	**<0.001**
		started aggression* vs. *reacted peacefully	**1.88**	**0.15**	**12.48**	**<0.001**
		reacted aggressively* vs. *reacted peacefully	**-0.99**	**0.27**	**-3.67**	**<0.001**
	Prevotella sp. - OTU 1887	***started aggression* vs. *reacted aggressively***	**2.86**	**0.24**	**12.14**	**<0.001**
		started aggression* vs. *reacted peacefully	**1.88**	**0.15**	**12.48**	**<0.001**
		reacted aggressively* vs. *reacted peacefully	**-0.99**	**0.27**	**-3.67**	**<0.001**
	Prevotella sp. OTU 20448	***started aggression* vs. *reacted aggressively***	**1.13**	**0.10**	**11.12**	**<0.001**
		started aggression* vs. *reacted peacefully	**0.74**	**0.09**	**8.38**	**<0.001**
		reacted aggressively* vs. *reacted peacefully	**-0.39**	**0.11**	**-3.42**	**0.002**
Pseudomonas	*Pseudomonas psychrotolerans* OTU 366	***started aggression* vs *reacted aggressively***	**0.27**	**0.13**	**2.08**	**0.095**
		started aggression* vs. *reacted peacefully	**1.09**	**0.17**	**6.33**	**<0.001**
		reacted aggressively* vs. *reacted peacefully	**0.82**	**0.18**	**4.57**	**<0.001**
	Pseudomonas sp. OTU 2442	***started aggression* vs. *reacted aggressively***	**1.15**	**0.21**	**5.50**	**<0.001**
		started aggression* vs. *reacted peacefully	**1.03**	**0.20**	**5.14**	**<0.001**
		reacted aggressively vs. *reacted peacefully*	-0.13	0.25	-0.50	0.871

Based on the multinomial logistic regressions, nine OTUs were associated with the behavioural states. We compared their counts in a generalised linear model and pairwise comparison with correction for multiple testing (see Main text for details). SE = standard error. Significant comparisons are shown in bold font.

Table S9. Intercept-only model vs all other models used in the multinomial logistic regression

Model used	Model comparison	Residual df	Residual deviance	df	LR statistics	p-value
1-8		162	178.96			
	**1 vs 2**	**156**	**141.95**	**6**	**37.01**	**<0.001**
	2 vs 3	154	157.93	2	-15.98	1.00
	3 vs 4	150	170.52	4	-12.59	1.00
	**4 vs 5**	**148**	**124.56**	**2**	**45.96**	**<0.001**
	5 vs 6	144	133.22	4	-8.66	1.00
	6 vs 7	142	140.24	2	-7.02	1.00
	**7 vs 8**	**136**	**111.61**	**6**	**28.63**	**<0.001**
9-16		162	178.96			
	**9 vs 10**	**156**	**178.96**	**6**	**37.01**	**<0.001**
	10 vs 11	156	141.95	0	-24.86	1.00
	**11 vs 12**	**150**	**166.81**	**6**	**36.53**	**<0.001**
	12 vs 13	150	130.28	0	-40.24	1.00
	**13 vs 14**	**144**	**170.52**	**6**	**37.30**	**<0.001**
	14 vs 15	144	133.22	0	-22.21	1.00
	**15 vs 16**	**138**	**120.24**	**6**	**35.20**	**<0.001**
17-24		162	178.96			
	**17 vs 18**	**156**	**141.95**	**6**	**37.01**	**<0.001**
	18 vs 19	154	157.93	2	-15.98	1.00
	19 vs 20	150	170.52	4	-12.59	1.00
	**20 vs 21**	**148**	**124.56**	**2**	**45.96**	**<0.001**
	21 vs 22	144	133.22	4	-8.66	1.00
	22 vs 23	142	140.24	2	-7.02	1.00
	**23 vs 24**	**136**	**111.61**	**6**	**28.62**	**<0.001**

df = degrees of freedom; LR statistics = likelihood ratio statistics. Bold columns represent statistically significant models. Model names have been altered because the order was changed in the analyses depending on the number of variables.

Model 1: Intercept only

Model 2: SNP1+SNP2 + SNP3

Model 3: Genes *CG3061* + *svr* + *Syt4* + *yellow-d2*

Model 4: Within-colony relatedness + TAS + PC1 CHC PCA + soil nitrogen values + mean annual precipitation + precipitation of the warmest quarter + mean annual temperature + maximum temperature of the warmest month

Model 5: SNP1+SNP2 + SNP3 + genes *CG3061* + *svr* + *Syt4* + *yellow-d2*

Model 6: SNP1+SNP2 + SNP3 + within-colony relatedness + TAS + PC1 CHC PCA + soil nitrogen values + mean annual precipitation + precipitation of the warmest quarter + mean annual temperature + maximum temperature of the warmest month

Model 7: Genes *CG3061* + *svr* + *Syt4* + *yellow-d2* + within-colony relatedness + TAS + PC1 CHC PCA + soil nitrogen values + mean annual precipitation + precipitation of the warmest quarter + mean annual temperature + maximum temperature of the warmest month

Model 8: SNP1+SNP2 + SNP3 + genes *CG3061* + *svr* + *Syt4* + *yellow-d2* + within-colony relatedness + TAS + PC1 CHC PCA + soil nitrogen values + mean annual precipitation + precipitation of the warmest quarter + mean annual temperature + maximum temperature of the warmest month

Model 9: Intercept only
Model 10: SNP1+SNP2 + SNP3
Model 11: Genes *CG3800* + *CG3902* + *CG34367*
Model 12: SNP1+SNP2 + SNP3 + genes CG3800 + CG3902 + CG34367
Model 13: Within-colony relatedness + TAS + PC1 CHC PCA + soil nitrogen values + mean annual precipitation + precipitation of the warmest quarter + mean annual temperature + maximum temperature of the warmest month
Model 14: SNP1+SNP2 + SNP3 + within-colony relatedness + TAS + PC1 CHC PCA + soil nitrogen values + mean annual precipitation + precipitation of the warmest quarter + mean annual temperature + maximum temperature of the warmest month
Model 15: Genes *CG3800* + *CG3902* + *CG34367*+ within-colony relatedness + TAS + PC1 CHC PCA + soil nitrogen values + mean annual precipitation + precipitation of the warmest quarter + mean annual temperature + maximum temperature of the warmest month
Model 16: SNP1+SNP2 + SNP3 + genes *CG3800* + *CG3902* + *CG34367*+ within-colony relatedness + TAS + PC1 CHC PCA + soil nitrogen values + mean annual precipitation + precipitation of the warmest quarter + mean annual temperature + maximum temperature of the warmest month
Model 17: Intercept only
Model 18: SNP1+SNP2 + SNP3
Model 19: *BicC* + *CG3238* + *Exo84* + *PlexA* + *CG3822* + *Rdl* + *RFC3*
Model 20: SNP1+SNP2 + SNP3 + *BicC* + *CG3238* + *Exo84* + *PlexA* + *CG3822* + *Rdl* + *RFC3*
Model 21: Within-colony relatedness + TAS + PC1 CHC PCA + soil nitrogen values + mean annual precipitation + precipitation of the warmest quarter + mean annual temperature + maximum temperature of the warmest month
Model 22: SNP1+SNP2 + SNP3 + within-colony relatedness + TAS + PC1 CHC PCA + soil nitrogen values + mean annual precipitation + precipitation of the warmest quarter + mean annual temperature + maximum temperature of the warmest month
Model 23: *BicC* + *CG3238* + *Exo84* + *PlexA* + *CG3822* + *Rdl* + *RFC3* + within-colony relatedness + TAS + PC1 CHC PCA + soil nitrogen values + mean annual precipitation + precipitation of the warmest quarter + mean annual temperature + maximum temperature of the warmest month
Model 24: SNP1+SNP2 + SNP3 + *BicC* + *CG3238* + *Exo84* + *PlexA* + *CG3822* + *Rdl* + *RFC3* + within-colony relatedness + TAS + PC1 CHC PCA + soil nitrogen values + mean annual precipitation + precipitation of the warmest quarter + mean annual temperature + maximum temperature of the warmest month

Table S10. AICc comparison for multinomial logistic regression Models 2-8, 10-16, and 18-24

Comparison	Models	K	AICc	ΔAICc	AICc Weight	Cumulative Weight	Log likelihood
Models 2-8	Model 2	8	159.92	0	0.92	0.92	-70.97
	Model 4	16	164.93	5.01	0.08	1	-62.28
	Model 3	10	181.02	21.1	0	1	-78.96
	Model 6	20	187.05	27.13	0	1	-66.64
	Model 8	28	198.26	38.34	0	1	-55.81
	Model 7	22	201.19	41.27	0	1	-70.02
	Model 5	14	204.32	44.4	0	1	-85
Models 10-16	Model 10	8	159.92	0	0.91	0.91	-70.97
	Model 12	14	164.55	4.63	0.09	1	-65.14
	Model 11	8	184.78	24.86	0	1	-83.4
	Model 14	20	186.99	27.07	0	1	-66.61
	Model 16	26	197.76	37.84	0	1	-60.12
	Model 13	14	204.79	44.86	0	1	-85.26
	Model 15	20	209.2	49.28	0	1	-77.72
Models 18-24	Model 20	22	157.95	0	0.73	0.73	-48.4
	Model 18	8	159.92	1.98	0.27	1	-70.97
	Model 19	16	174.06	16.11	0	1	-66.84
	Model 22	20	186.99	29.05	0	1	-66.61
	Model 23	28	201.58	43.64	0	1	-57.47
	Model 24	34	204.67	46.73	0	1	-43.02
	Model 21	14	204.79	46.84	0	1	-85.26

K=number of parameters used in the model; Models highlighted in grey represent the two best models based on Akaike Information Criterion for small sample sizes (AICc) and ΔAICc. Note that Models 1, 9, and 17 are not presented as they are the intercept-only models.

Table S11. Results of multinomial logistic regression Model 2 compared with the baseline *started aggression*

Behaviour	SNPs	Estimate	log_n odds ratio	SE	Z-statistics	p-value	Lower CI	Upper CI	Log-likelihood LRT	df LRT	χ^2 value LRT	p-value LRT	significance LRT
Reacted aggressively	Intercept	-3.11	0.05	2.59	-1.20	0.231							
	SNP1	0.24	1.27	1.10	0.22	0.830	0.15	10.90	-71.02	2	0.081	0.960	
	**SNP2**	**3.45**	**31.54**	**1.15**	**3.01**	**0.003**	**3.33**	**299.00**	**-80.44**	**2**	**18.93**	**<0.001**	***
	**SNP3**	**-2.46**	**0.09**	**0.71**	**-3.44**	**<0.001**	**0.02**	**0.35**	**-78.86**	**2**	**15.77**	**<0.001**	***
Reacted peacefully	Intercept	-1.73	0.18	2.16	-0.80	0.42							
	SNP1	-0.02	0.98	1.12	-0.01	0.988	0.11	8.79	-71.02	2	0.081	0.960	
	**SNP2**	**2.47**	**11.78**	**0.89**	**2.78**	**0.006**	**2.06**	**67.20**	**-80.44**	**2**	**18.93**	**<0.001**	***
	**SNP3**	**-1.95**	**0.14**	**0.69**	**-2.84**	**<0.001**	**0.04**	**0.55**	**-78.86**	**2**	**15.77**	**<0.001**	***

LRT = likelihood ratio test. Asterisks denote statistical p-values with *** = p-value <0.001. SE = standard error. CI = Confidence Interval. Statistically significant genes are represented in bold letters.

Table S12. Results of multinomial logistic regression Model 4 compared with the baseline *started aggression*

**Behaviour**	**SNPs or DEGs**	**Estimate**	**$\log_n$ odds ratio**	**SE**	**Z-statistics**	**p-value**	**lower CI**	**upper CI**	**Log-likelihood LRT**	**df LRT**	**χ^2 value LRT**	**p-value LRT**	**significance LRT**
Reacted aggressively	Intercept	-4.06	0.02	0.11	-37.84								
	SNP1	0.19	1.22	0.46	0.42		0.49	3.02	-62.54	2	0.53	0.769	
	**SNP2**	**3.67**	**39.21**	**0.32**	**11.51**	***	**20.99**	**73.23**	**-70.52**	**2**	**16.49**	**<0.001**	***
	**SNP3**	**-2.83**	**0.06**	**0.38**	**7.46**	***	**0.03**	**0.12**	**-69.80**	**2**	**15.03**	**<0.001**	***
	CG3061	0.00	1.00	0.01	0.01		0.98	1.02	-62.28	2	<0.001	0.999	
	silver	0.00	1.00	00	0.67		0.99	1.01	-62.76	2	0.95	0.621	
	Syt4	-0.01	0.99	0.00	-1.22		0.99	1.00	-63.07	2	1.58	0.454	
	yellow-d2	**0.01**	**1.00**	**0.00**	**2.15**	*	**1.00**	**1.01**	**-65.91**	**2**	**7.26**	**0.027**	*
Reacted peacefully	Intercept	-1.07	0.34	0.11	-9.37								
	SNP1	-0.53	0.59	0.48	-1.09		0.23	1.52	-62.54	2	0.53	0.769	
	SNP2	**2.46**	11.76	**0.40**	**6.09**	***	**5.32**	**26.00**	**-70.52**	**2**	**16.49**	**<0.001**	***
	SNP3	**-1.96**	0.14	**0.41**	**4.81**	***	**0.06**	**0.31**	**-69.80**	**2**	**15.03**	**<0.001**	***
	CG3061	0.00	1.00	0.01	0.02		0.98	1.02	-62.28	2	<0.001	0.999	
	silver	0.00	1.00	00	0.12		0.99	1.01	-62.76	2	0.95	0.621	
	Syt4	0.00	1.00	0.00	-0.82		0.99	1.01	-63.07	2	1.58	0.454	
	yellow-d2	**0.01**	**1.00**	**0.00**	**2.36**	*	**1.00**	**1.01**	**-65.91**	**2**	**7.26**	**0.027**	*

SNPs = Single Nucleotide Polymorphism, DEGs = Differentially expressed genes, LRT = likelihood ratio test. SE = standard error. CI = Confidence Interval. χ2 value LRT = the Chi-Squared value of the LRT test, Asterisks denote statistical p-values with *** = p-value <0.001 and * = p-value <0.05. Statistically significant genes are represented in bold letters.

Table S13. Nagelkerke Pseudo-R^2 of all multinomial logistic regression models

Model comparisons	Model	Nagelkerke Pseudo-R^2
Models 2-8	**Model 2**	**0.41**
	**Model 4**	**0.55**
	Model 3	0.26
	Model 6	0.48
	Model 8	0.63
	Model 7	0.42
	Model 5	0.11
Models 10-16	Model 10	0.41
	**Model 12**	**0.50**
	Model 11	0.16
	Model 14	0.48
	Model 16	0.58
	Model 13	0.11
	Model 15	0.28
Models 18-24	**Model 20**	**0.71**
	Model 18	0.41
	Model 19	0.48
	Model 22	0.48
	Model 23	0.61
	Model 24	0.76
	Model 21	0.11

Note that the order of the Models follows the Akaike Information Criterion for small sample sizes (AICc) comparison. Models 1, 9, and 17 are not included as they are the intercept model. Models highlighted in grey are the best models and models in bold font are the models selected for further inspection.

Table S14. Emmeans contrasts for Models 2, 4, 12, and 20

Model	Contrast	Estimate	SE	df	t-ratio	p-value
	Started aggression vs. *reacted aggressively*	0.3302	0.128	8	2.590	0.0741
2	*Started aggression* vs. *reacted peacefully*	0.3533	0.137	8	2.579	0.0753
	Reacted aggressively vs. *reacted peacefully*	0.0231	0.111	8	0.208	0.9765
	Started aggression vs. *reacted aggressively*	0.235	0.112	16	2.098	0.122
4	*Started aggression* vs. *reacted peacefully*	0.270	0.126	16	2.137	0.114
	Reacted aggressively vs. *reacted peacefully*	0.035	0.125	16	0.276	0.958
	Started aggression vs. *reacted aggressively*	0.341	0.138	14	2.472	0.065
12	***Started aggression* vs. *reacted peacefully***	**0.378**	**0.134**	**14**	**2.818**	**0.034***
	Reacted aggressively vs. *reacted peacefully*	0.037	0.116	14	0.319	0.958
	Started aggression* vs. *reacted aggressively	**-0.341**	**-0.057**	**22**	**-5.984**	**<0.001*** **
20	***Started aggression* vs. *reacted peacefully***	**-0.479**	**-0.069**	**22**	**-6.923**	**<0.001*** **
	Reacted aggressively vs. *reacted peacefully*	-0.138	0.113	22	-1.225	0.451

Note: SE = standard error. df= degrees of freedom. Statistically significant comparisons are highlighted in bold letters. Ssterisks denote statistical p-values with *** = p-value <0.001 and * = p-value <0.05.

Table S15. Log-likelihood ratio tests for Models 2, 4, 12, and 20

Model	Variable	Log-likelihood	df	χ2 test value	p-value
Model2	SNP1	-71.015	6	0.0814	0.960
	**SNP2**	**-80.437**	**6**	**18.925**	**<0.001*****
	**SNP3**	**-78.860**	**6**	**15.77**	**<0.001*****
Model4	SNP1	-62.542	14	0.5263	0.769
	**SNP2**	**-70.524**	**14**	**16.489**	**<0.001*****
	**SNP3**	**-29.795**	**14**	**15.031**	**<0.001*****
	Gene *CG3061*	-62.280	14	0.0006	0.999
	Gene *silver*	-62.755	14	0.9518	0.6213
	Gene *Syt*	-63.069	14	1.5787	0.4541
	**Gene *yellow-d2***	**-65,910**	**14**	**7.2606**	**0.0265***
Model 12	SNP1	-65.220	12	0.1609	0.923
	**SNP2**	**-73.898**	**12**	**17.517**	**<0.001*****
	**SNP3**	**-73.060**	**12**	**15.841**	**<0.001*****
	Gene *CNBP*	-66.415	12	2.551	0.279
	Gene *CG3902*	-66.079	12	1.880	0.391
	**Gene *CG34367***	**-68.754**	**12**	**7.230**	**0.029***
Model 20	SNP1	-48.427	20	0.061	0.970
	**SNP2**	**-55.193**	**20**	**13.593**	**0.001****
	**SNP3**	**-60.409**	**20**	**24.024**	**<0.001*****
	**Gene *BicC***	**-53.104**	**20**	**9.415**	**0.009****
	**Gene *Pif1***	**-52.749**	**20**	**8.705**	**0.0129***
	Gene *Exo84*	-49.617	20	2.441	0.295
	Gene *PlexA*	-48.940	20	1.086	0.581
	Gene *KaiR1d*	-50.055	20	3.318	0.190
	Gene *Rdl*	-49.523	20	2.253	0.324
	**Gene *RFC3***	**-51.905**	**20**	**7.017**	**0.029***

Note: df= degrees of freedom. χ2 test value = Chi-Squared test value. Asterisks denote p-values with *** = p-value <0.001, ** = p-value <0.01, and * = p-value <0.05. df= degrees of freedom. Statistically significant Single Nucleotide Polymorphisms (SNPs) and differentially-expresses genes (DEGs) are represented in bold letters.

Table S16. Results of multinomial logistic regression Model 12 compared with the baseline *started aggression*

Behaviour	SNP or DEG	Estimate	log_n odds ratio	SE	Z-statistics	p-value	Lower CI	Upper CI	Log-likelihood LRT	df LRT	χ^2 value LRT	p-value LRT	significance LRT
Reacted aggressively	Intercept	-4.04		2.66	-1.52								
	SNP1	0.32	1.37	1.21	0.26	0.792	0.13	14.59	-65.22	2	0.16	0.923	
	**SNP2**	**3.47**	**32.05**	**1.19**	**2.92**	**0.003**	**3.13**	**327.64**	**-73.90**	**2**	**17.52**	**0.000**	***
	**SNP3**	**-2.57**	**0.08**	**0.79**	**-3.26**	**0.001**	**0.02**	**0.36**	**-73.06**	**2**	**15.84**	**0.000**	***
	CG3800	0.00	1.00	0.00	-0.74	0.460	0.99	1.00	-66.42	2	2.55	0.279	
	CG3902	-0.01	0.99	0.02	-0.49	0.622	0.96	1.02	-66.08	2	1.88	0.391	
	CG34367	**0.06**	**1.07**	**0.04**	**1.58**	**0.114**	**0.98**	**1.15**	**-68.75**	**2**	**7.23**	**0.027**	*
Reacted peacefully	Intercept	-1.69		2.30	-0.74								
	SNP1	-0.11	0.90	1.17	-0.09	0.927	0.09	8.95	-65.22	2	0.16	0.923	
	SNP2	**2.43**	**11.37**	**0.91**	**2.66**	**0.008**	**1.89**	**68.32**	**-73.90**	**2**	**17.52**	**0.000**	***
	SNP3	**-2.20**	**0.11**	**0.75**	**-2.95**	**0.003**	**0.03**	**0.48**	**-73.06**	**2**	**15.84**	**0.000**	***
	CG3800	0.00	1.00	0.00	0.70	0.485	1.00	1.00	-66.42	2	2.55	0.279	
	CG3902	0.01	1.01	0.02	0.81	0.416	0.98	1.05	-66.08	2	1.88	0.391	
	CG34367	**-0.02**	**0.98**	**0.04**	**-0.43**	**0.665**	**0.91**	**1.06**	**-68.75**	**2**	**7.23**	**0.027**	*

SNPs = Single Nucleotide Polymorphism, DEGs = Differentially expressed genes, LRT = likelihood ratio test. SE = standard error. CI = Confidence Interval. $\chi2$ value LRT = the Chi-Squared value of the LRT test, Asterisks denote statistical p-values with *** = p-value <0.001 and * = p-value <0.05. Statistically significant genes are represented in bold letters.

Table S17. Results of the multinomial logistic regression Model 20 compared with the baseline *started aggression*

Behaviour	SNP or DEG	Estimate	$\log_n$ odds ratio	SE	Z-statistics	p-value	Lower CI	Upper CI	Log-likelihood LRT	df LRT	χ^2 value LRT	p-value LRT	significance LRT
Reacted aggressively	Intercept	-4.66		0.00	-5550.79								
	SNP1	0.44	53.90	0.00	523.24	<0.001	1.55	1.56	-48.43	2	0.06	0.970	
	**SNP2**	**3.99**	**0.01**	**0.00**	**2534.23**	**<0.001**	**53.73**	**54.06**	**-55.19**	**2**	**13.59**	**0.001**	**
	**SNP3**	**-4.94**	**1.94**	**0.00**	**-3885.30**	**<0.001**	**0.01**	**0.01**	**-60.41**	**2**	**24.02**	**0.000**	***
	BicC	**0.66**	**1.25**	**0.01**	**120.22**	**<0.001**	**1.92**	**1.97**	**-53.10**	**2**	**9.41**	**0.009**	**
	Pif1	**0.23**	**1.00**	**0.00**	**132.92**	**<0.001**	**1.25**	**1.26**	**-52.75**	**2**	**8.71**	**0.013**	*
	Exo84	**0.00**	**1.00**	**0.00**	**-1.99**	**0.046**	**1.00**	**1.00**	**-49.62**	**2**	**2.44**	**0.295**	
	PlexA	0.00	1.12	0.03	-0.02	0.984	0.95	1.06	-48.94	2	1.09	0.581	
	Kai1R1d	**0.11**	**1.00**	**0.03**	**3.33**	**0.001**	**1.05**	**1.19**	**-50.06**	**2**	**3.32**	**0.190**	
	Rdl	0.00	1.29	0.00	-1.05	0.294	1.00	1.00	-49.52	2	2.25	0.324	
	RFC3	**0.25**	**53.90**	**0.02**	**15.12**	**<0.001**	**1.25**	**1.33**	**-51.90**	**2**	**7.02**	**0.030**	*
Reacted peacefully	Intercept	-3.73		0.00	-4450.71								
	SNP1	0.45	1.56	0.00	**494.85**	<0.001	1.56	1.56	-48.43	2	0.06	0.970	
	**SNP2**	**3.44**	**31.03**	**0.00**	**2261.55**	**<0.001**	**30.94**	**31.12**	**-55.19**	**2**	**13.59**	**0.001**	**
	**SNP3**	**-4.17**	**0.02**	**0.00**	**-3082.30**	**<0.001**	**0.02**	**0.02**	**-60.41**	**2**	**24.02**	**0.000**	***
	BicC	**0.66**	**1.93**	**0.01**	**118.51**	**<0.001**	**1.91**	**1.95**	**-53.10**	**2**	**9.41**	**0.009**	**
	Pif1	**-0.12**	**0.89**	**0.00**	**-37.90**	**<0.001**	**0.89**	**0.90**	**-52.75**	**2**	**8.71**	**0.013**	*
	Exo84	**0.00**	**1.00**	**0.00**	**-2.08**	**0.038**	**1.00**	**1.00**	**-49.62**	**2**	**2.44**	**0.295**	
	PlexA	-0.03	0.97	0.03	-0.92	0.356	0.92	1.03	-48.94	2	1.09	0.581	
	Kai1R1d	**0.18**	**1.20**	**0.03**	**5.45**	**<0.001**	**1.13**	**1.29**	**-50.06**	**2**	**3.32**	**0.190**	
	Rdl	0.00	1.00	0.00	0.00	0.997	1.00	1.00	-49.52	2	2.25	0.324	
	RFC3	**0.24**	**1.27**	**0.02**	**14.20**	**<0.001**	**1.23**	**1.31**	**-51.90**	**2**	**7.02**	**0.030**	*

SNPs = Single Nucleotide Polymorphism, DEGs = Differentially expressed genes, LRT = likelihood ratio test. SE = standard error. CI = Confidence Interval. $\chi 2$ value LRT = the Chi-Squared value of the LRT test, Asterisks denote statistical p-values with *** = p-value <0.001 ** = p-value <0.01, and * = p-value <0.05. Statistically significant genes are represented in bold letters.

Table S18. Results of the final multinomial logistic regression model comparing the baseline behaviour *started aggression* with *reacted aggressively* and *reacted peacefully* including log-likelihood ratio tests

Behaviour	SNP or DEG	Estimate	log_n odds ratio	SE	Z-statistics	p-value	lower CI	upper CI	Log-likelihood LRT	df LRT	χ^2 value LRT	p-value LRT	significance LRT
Reacted aggressively	**SNP1**	**133.76**	**4.90**	**0.00**	**3240.82**	**<0.001**	**133.36**	**134.15**	**-52.12**	**20**	**15.01**	**0.001**	***
	**SNP3**	**0.02**	**-4.13**	**0.00**	**-3210.27**	**<0.001**	**0.02**	**0.02**	**-52.12**	**20**	**15.01**	**0.001**	***
	yellow-d2	**1.03**	**0.03**	**0.01**	**2.69**	**0.007**	**1.01**	**1.05**	**-48.43**	**20**	**7.63**	**0.022**	*
	BicC	**1.79**	**0.58**	**0.01**	**102.90**	**<0.001**	**1.77**	**1.81**	**-48.84**	**20**	**8.45**	**0.015**	*
	Pif1	**1.41**	**0.34**	**0.00**	**168.31**	**<0.001**	**1.40**	**1.41**	**-48.95**	**20**	**8.67**	**0.013**	*
	Exo84	**1.00**	**-0.0002**	**0.00**	**-3.73**	**0.000**	**1.00**	**1.00**	**-47.68**	**20**	**6.13**	**0.047**	*
	PlexA	1.02	0.02	0.03	0.57	0.567	0.96	1.08	-45.15	20	1.07	0.585	ns
	KaiR1d	**1.36**	**0.31**	**0.04**	**8.54**	**0.000**	**1.27**	**1.46**	**-47.99**	**20**	**6.75**	**0.034**	*
	Rdl	**1.00**	**0.00**	**0.00**	**-3.47**	**0.001**	**1.00**	**1.00**	**-48.88**	**20**	**8.53**	**0.014**	*
	RFC3	**1.37**	**0.32**	**0.02**	**16.80**	**0.000**	**1.32**	**1.42**	**-51.03**	**20**	**12.83**	**0.002**	**
Reacted peacefully	**SNP1**	**44.58**	**3.80**	**0.00**	**2647.91**	**<0.001**	**44.45**	**44.70**	**-52.12**	**20**	**15.01**	**0.001**	***
	**SNP3**	**0.02**	**-3.81**	**0.00**	**-3207.65**	**<0.001**	**0.02**	**0.02**	**-48.43**	**20**	**15.01**	**0.001**	***
	yellow-d2	**1.02**	**0.02**	**0.01**	**2.21**	**0.027**	**1.00**	**1.04**	**-48.84**	**20**	**7.63**	**0.022**	*
	BicC	**1.77**	**0.57**	**0.01**	**101.86**	**<0.001**	**1.75**	**1.79**	**-48.95**	**20**	**8.45**	**0.015**	*
	Pif1	**1.00**	**0.001**	**0.00**	**0.22**	**0.828**	**0.99**	**1.01**	**-47.68**	**20**	**8.67**	**0.013**	*
	Exo84	**1.00**	**-0.0002**	**0.00**	**-3.43**	**0.001**	**1.00**	**1.00**	**-45.15**	**20**	**6.13**	**0.047**	*
	PlexA	0.99	-0.01	0.03	-0.36	0.716	0.93	1.05	-47.99	20	1.07	0.585	ns
	KaiR1d	**1.43**	**0.36**	**0.04**	**10.20**	**0.000**	**1.34**	**1.53**	**-48.88**	**20**	**6.75**	**0.034**	*
	Rdl	**1.00**	**-0.002**	**0.00**	**-2.59**	**0.009**	**1.00**	**1.00**	**-51.03**	**20**	**8.53**	**0.014**	*
	RFC3	**1.33**	**0.29**	**0.02**	**15.31**	**0.000**	**1.28**	**1.38**	**-52.12**	**20**	**12.83**	**0.002**	**

SNPs = Single Nucleotide Polymorphism, DEGs = Differentially expressed genes, LRT = likelihood ratio test. SE = standard error. CI = Confidence Interval. $\chi 2$ value LRT = the Chi-Squared value of the LRT test, Asterisks denote statistical p-values with *** = p-value <0.001 ** = p-value <0.01, and * = p-value <0.05. Statistically significant genes are represented in bold letters.

Table S19. Gene-ontology data for the differentially expressed genes and the SNP3 gene *gd*

Source	Term name	Gene Ontology Term ID	Adjusted p-value	Negative log_{10} of adjusted p-value	Term size	Query size	Intersections
MF	transmitter-gated monoatomic ion channel activity	GO: 0022824	0.009	2.039	45	8	*KaiR1D, Rdl*
MF	cyclase activator activity	GO:0010853	0.015	1.835	3	8	*PlexA*
MF	semaphorin receptor activity	GO:0017154	0.015	1.835	2	8	*PlexA*
MF	14-3-3 protein binding	GO:0071889	0.015	1.835	3	8	*PlexA*
MF	ATP-dependent activity, acting on DNA	GO:0008094	0.015	1.835	74	8	*Pif1, RFC3*
MF	5'-3' DNA helicase activity	GO:0043139	0.017	1.761	4	8	*Pif1*
BP	sensory neuron axon guidance	GO:0097374	0.045	1.348	5	8	*PlexA*
BP	calcium ion import across plasma membrane	GO:0098703	0.045	1.348	6	8	*KaiR1D*
BP	semaphorin-plexin signaling pathway involved in axon guidance	GO:1902287	0.045	1.348	2	8	*PlexA*
BP	semaphorin-plexin signaling pathway involved in regulation of photoreceptor cell axon guidance	GO:2000305	0.045	1.348	2	8	*PlexA*
BP	DNA replication	GO:0006260	0.045	1.348	134	8	*Pif1, RFC3*
BP	positive regulation of neurotransmitter secretion	GO:0001956	0.045	1.348	6	8	*KaiR1D*
BP	positive regulation of guanylate cyclase activity	GO:0031284	0.045	1.348	2	8	*PlexA*
BP	regulation of membrane potential	GO:0042391	0.045	1.348	102	8	*KaiR1D, Rdl*
BP	leading strand elongation	GO:0006272	0.049	1.314	8	8	*RFC3*
BP	synaptic vesicle targeting	GO:0016080	0.049	1.314	7	8	*Exo84*
BP	negative regulation of oskar mRNA translation	GO:0007319	0.049	1.314	8	8	*BicC*
BP	positive regulation of neuromuscular synaptic transmission	GO:1900075	0.049	1.314	8	8	*KaiR1D*
CC	replication fork	GO:0005657	0.018	1.735	35	9	*Pif1, RFC3*
CC	semaphorin receptor complex	GO:0002116	0.018	1.735	2	9	*PlexA*
CC	postsynaptic membrane	GO:0045211	0.018	1.735	71	9	*KaiR1D, Rdl*

CC	GABA receptor complex	GO:1902710	0.018	1.735	2	9	*Rdl*
CC	neuronal cell body membrane	GO:0032809	0.020	1.693	4	9	*Rdl*
CC	Elg1 RFC-like complex	GO:0031391	0.020	1.693	5	9	*RFC3*
CC	exocyst	GO:0000145	0.029	1.536	8	9	*Exo84*
CC	chloride channel complex	GO:0034707	0.041	1.384	13	9	*Rdl*

MF = Molecular function, BP = Biological processes, CC = Cellular components, GO = Gene Ontology. Retrieved on 26.12.2025 from g:Profiler (https://biit.cs.ut.ee/gprofiler/gost).

Table S20. List of the SNP and DEGs used in the GeneMania analysis

Gene list	Function	Overall function	FDR-corrected p-value	Genes in network	Genes in genome
BicC, gd, KaiR1d, PlexA, Rdl, yellow-d2, Exo84, Pif1, RFC3	**nuclear chromosome segregation**	**Biological processes**	**0.004**	**6**	**161**
	**DNA metabolic process**	**Biological processes**	**0.004**	**7**	**271**
	**extracellular ligand-gated ion channel activity**	**Molecular functions**	**0.004**	**4**	**35**
	**chromosome segregation**	**Biological processes**	**0.004**	**6**	**165**
	**DNA recombination**	**Biological processes**	**0.004**	**5**	**94**
	**meiosis I cell cycle process**	**Biological processes**	**0.007**	**5**	**108**
	**ligand-gated ion channel activity**	**Molecular functions**	**0.048**	**4**	**80**
	transmitter-gated ion channel activity		0.053	3	30
	transmitter-gated channel activity		0.053	3	30
	meiotic cell cycle process		0.088	5	202
	meiotic cell cycle		0.088	5	208
	neurotransmitter receptor activity		0.088	3	39
BicC, KaiR1d, PlexA, Rdl, yellow-d2, Exo84, Pif1, RFC3 (without *gd*)	**extracellular ligand-gated ion channel activity**	**Molecular functions**	**0.010**	**4**	**35**
	**trans-synaptic signaling**	**Biological processes**	**0.040**	**6**	**287**
	**nuclear chromosome segregation**	**Biological processes**	**0.040**	**5**	**161**
	**synaptic signaling**	**Biological processes**	**0.040**	**6**	**288**
	**DNA metabolic process**	**Biological processes**	**0.040**	**6**	**271**
	**chromosome segregation**	**Biological processes**	**0.040**	**5**	**165**
	**anterograde trans-synaptic signaling**	**Biological processes**	**0.040**	**6**	**286**
	**ligand-gated ion channel activity**	**Molecular functions**	**0.040**	**4**	**80**
	**transmitter-gated ion channel activity**	**Molecular functions**	**0.043**	**3**	**30**
	**transmitter-gated channel activity**	**Molecular functions**	**0.043**	**3**	**30**
	DNA recombination		0.050	4	94
	neurotransmitter receptor activity		0.079	3	39

SNPs = Single Nucleotide Polymorphism, DEGs = Differentially expressed genes, FDR = false-discovery rate. Bold font represents significant DEGs after FDR-correction.

SI References

1. Dobin, A., Davis, C.A., Schlesinger, F., Drenkow, J., Zaleski, C., Jha, S., Batut, P., Chaisson, M., and Gingeras, T.R. (2012). STAR: ultrafast universal RNA-seq aligner. Bioinformatics *29*, 15-21. https://doi.org/10.1093/bioinformatics/bts635.
2. Steiner, F.M., Schlick-Steiner, B.C., Moder, K., Stauffer, C., Arthofer, W., Buschinger, A., Espadaler, X., Christian, E., Einfinger, K., Lorbeer, E., et al. (2007). Abandoning aggression but maintaining self-nonself discrimination as a first stage in ant supercolony formation. Curr Biol *17*, 1903-1907. https://doi.org/10.1016/j.cub.2007.09.061
3. Davidson, S., Shanley, L., Cowie, P., Lear, M., McGuffin, P., Quinn, J.P., Barrett, P., and MacKenzie, A. (2016). Analysis of the effects of depression associated polymorphisms on the activity of the BICC1 promoter in amygdala neurones. Pharmacogenomics J *16*, 366-374. https://doi.org/10.1038/tpj.2015.62.
4. Ota, K.T., Andres, W., Lewis, D.A., Stockmeier, C.A., and Duman, R.S. (2015). BICC1 expression is elevated in depressed subjects and contributes to depressive behavior in rodents. Neuropsychopharmacology *40*, 711-718. https://doi.org/10.1038/npp.2014.227.
5. Ryan, J., Artero, S., Carrière, I., Maller, J.J., Meslin, C., Ritchie, K., and Ancelin, M.-L. (2016). GWAS-identified risk variants for major depressive disorder: Preliminary support for an association with late-life depressive symptoms and brain structural alterations. Eur Neuropsychopharmacol *26*, 113-125. https://doi.org/10.1016/j.euroneuro.2015.08.022.
6. Kraus, M.R.-C., Clauin, S., Pfister, Y., Di Maïo, M., Ulinski, T., Constam, D., Bellanné-Chantelot, C., and Grapin-Botton, A. (2012). Two mutations in human BICC1 resulting in Wnt pathway hyperactivity associated with cystic renal dysplasia. Hum Mutat *33*, 86-90. https://doi.org/10.1002/humu.21610.
7. Komiya, Y., and Habas, R. (2008). Wnt signal transduction pathways. Organogenesis *4*, 68-75. https://doi.org/10.4161/org.4.2.5851.
8. Vega, I.E., and Hsu, S.-C. (2001). The Exocyst complex associates with microtubules to mediate vesicle targeting and neurite outgrowth. J Neurosci *21*, 3839-3848. https://doi.org/10.1523/jneurosci.21-11-03839.2001.
9. Hsu, S.-C., Ting, A.E., Hazuka, C.D., Davanger, S., Kenny, J.W., Kee, Y., and Scheller, R.H. (1996). The mammalian brain rsec6/8 complex. Neuron *17*, 1209-1219. https://doi.org/10.1016/S0896-6273(00)80251-2.
10. Blankenship, J.T., Fuller, M.T., and Zallen, J.A. (2007). The *Drosophila* homolog of the Exo84 exocyst subunit promotes apical epithelial identity. J Cell Sci *120*, 3099-3110. https://doi.org/10.1242/jcs.004770.
11. Tanaka, T., Goto, K., and Iino, M. (2017). Diverse functions and signal transduction of the Exocyst complex in tumor cells. J Cell Physiol *232*, 939-957. https://doi.org/10.1002/jcp.25619.
12. Kiragasi, B., Wondolowski, J., Li, Y., and Dickman, D.K. (2017). A presynaptic glutamate receptor subunit confers robustness to neurotransmission and homeostatic potentiation. Cell Reports *19*, 2694-2706. https://doi.org/10.1016/j.celrep.2017.06.003.
13. Liu, X., Krause, W.C., and Davis, R.L. (2007). $GABA_A$ receptor RDL inhibits *Drosophila* olfactory associative learning. Neuron *56*, 1090-1102. https://doi.irg/10.1016/j.neuron.2007.10.036.

14. Yuan, Q., Song, Y., Yang, C.-H., Jan, L.Y., and Jan, Y.N. (2014). Female contact modulates male aggression via a sexually dimorphic GABAergic circuit in *Drosophila*. Nat Neurosci *17*, 81-88. https://doi.org/10.1038/nn.3581.
15. Li, J., and Christensen, B.M. (2011). Biological function of insect yellow gene family. In Recent advances in entomological research: from molecular biology to pest management, T. Liu, and L. Kang, eds. (Beijing and Springer-Verlag Berlin Heidelberg), pp. 121-131. Isbn: 3642178146.
16. Drapeau, M.D., Radovic, A., Wittkopp, P.J., and Long, A.D. (2003). A gene necessary for normal male courtship, yellow, acts downstream of fruitless in the *Drosophila melanogaster* larval brain. J Neurobiol *55*, 53-72. https://doi.org/10.1002/neu.10196.
17. Drapeau, M.D. (2003). A novel hypothesis on the biochemical role of the *Drosophila* Yellow protein. Biochem Biophys Res Com *311*, 1-3. https://doi.org/10.1016/j.bbrc.2003.09.106.
18. Tamagnone, L., Artigiani, S., Chen, H., He, Z., Ming, G.-l., Song, H.-j., Chedotal, A., Winberg, M.L., Goodman, C.S., Poo, M.-m., et al. (1999). Plexins are a large family of receptors for transmembrane, secreted, and gpi-anchored semaphorins in vertebrates. Cell *99*, 71-80. https://doi.org/10.1016/S0092-8674(00)80063-X.
19. Fisher, H.P., Pascual, M.G., Jimenez, S.I., Michaelson, D.A., Joncas, C.T., Quenzer, E.D., Christie, A.E., and Horch, H.W. (2018). De novo assembly of a transcriptome for the cricket *Gryllus bimaculatus* prothoracic ganglion: An invertebrate model for investigating adult central nervous system compensatory plasticity. PLoS ONE *13*, e0199070. https://doi.org/10.1371/journal.pone.0199070.
20. Duan, Y., Wang, S.-H., Song, J., Mironova, Y., Ming, G.-l., Kolodkin, A.L., and Giger, R.J. (2014). Semaphorin 5A inhibits synaptogenesis in early postnatal- and adult-born hippocampal dentate granule cells. eLife *3*, e04390. https://doir.org/10.7554/eLife.04390.
21. Chung, W.-H. (2014). To peep into Pif1 helicase: Multifaceted all the way from genome stability to repair-associated DNA synthesis. J Microbiol *52*, 89-98. https://doi.org/10.1007/s12275-014-3524-3.
22. Byrd, A.K., and Raney, K.D. (2017). Structure and function of Pif1 helicase. Biochem Soc Trans *45*, 1159-1171. https://doi.org/10.1042/bst20170096.
23. Li, X., and Burgers, P.M. (1994). Molecular cloning and expression of the Saccharomyces cerevisiae RFC3 gene, an essential component of replication factor C. Proc Natl Acad Sci *91*, 868-872. https://doi.org/10.1073/pnas.91.3.868.
24. Zhou, J., Wen-Wen, Z., Fang, P., Jia-Yuan, S., Zhen-Yu, H., and and Wu, S.-G. (2018). Downregulation of hsa_circ_0011946 suppresses the migration and invasion of the breast cancer cell line MCF-7 by targeting RFC3. Cancer Manag Res *10*, 535-544. https://doi.org/10.2147/CMAR.S155923.
25. Queller, D.C., and Goodnight, K.F. (1989). Estimating relatedness using genetic-markers. Evolution *43*, 258-275. https://doi.org/10.2307/2409206.
26. Pamilo, P. (1991). Evolution of colony characteristics in social insects. II. Number of reproductive individuals. Am Nat *138*, 412-433. https://www.jstor.org/stable/2462158.

27. Manichaikul, A., Mychaleckyj, J.C., Rich, S.S., Daly, K., Sale, M., and Chen, W.-M. (2010). Robust relationship inference in genome-wide association studies. Bioinformatics *26*, 2867-2873. https://doi.org/10.1093/bioinformatics/btq559.
